# Induced pluripotent stem cells of patients with Tetralogy of Fallot reveal transcriptional alterations in cardiomyocyte differentiation

**DOI:** 10.1038/s41598-020-67872-z

**Published:** 2020-07-02

**Authors:** Marcel Grunert, Sandra Appelt, Sophia Schönhals, Kerstin Mika, Huanhuan Cui, Ashley Cooper, Lukas Cyganek, Kaomei Guan, Silke R. Sperling

**Affiliations:** 1 Cardiovascular Genetics, Charité - Universitätsmedizin Berlin, Berlin, Germany; 2 Berlin Institute of Health (BIH), Berlin, Germany; 3 DZHK (German Centre for Cardiovascular Research), Partner Site Berlin, Berlin, Germany; 4 Department of Biology, Chemistry, and Pharmacy, Freie Universität Berlin, Berlin, Germany; 5 Institute of Pharmacology and Toxicology, University Medical Center Göttingen, Göttingen, Germany; 6 Stem Cell Unit, Clinic for Cardiology and Pneumology, University Medical Center Göttingen, Göttingen, Germany; 7 Department of Cardiology and Pneumology, University Medical Center Göttingen, Göttingen, Germany; 8 Institute of Pharmacology and Toxicology, Technische Universität Dresden, Dresden, Germany

**Keywords:** Congenital heart defects, Transcriptomics, Gene expression analysis, Next-generation sequencing, Induced pluripotent stem cells, Statistical methods, Disease model, Gene regulatory networks

## Abstract

Patient-specific induced pluripotent stem cells (ps-iPSCs) and their differentiated cell types are a powerful model system to gain insight into mechanisms driving early developmental and disease-associated regulatory networks. In this study, we use ps-iPSCs to gain insights into Tetralogy of Fallot (TOF), which represents the most common cyanotic heart defect in humans. iPSCs were generated and further differentiated to cardiomyocytes (CMs) using standard methods from two well-characterized TOF patients and their healthy relatives serving as controls. Patient-specific expression patterns and genetic variability were investigated using whole genome and transcriptome sequencing data. We first studied the clonal mutational burden of the derived iPSCs. In two out of three iPSC lines of patient TOF-01, we found a somatic mutation in the DNA-binding domain of tumor suppressor P53, which was not observed in the genomic DNA from blood. Further characterization of this mutation showed its functional impact. For patient TOF-02, potential disease-relevant differential gene expression between and across cardiac differentiation was shown. Here, clear differences at the later stages of differentiation could be observed between CMs of the patient and its controls. Overall, this study provides first insights into the complex molecular mechanisms underlying iPSC-derived cardiomyocyte differentiation and its transcriptional alterations in TOF.

## Introduction

Malformations of the heart represent the most common birth defect in human with an incidence of nearly 1% of all live births^1,2^. There is a long and remarkable history of clinical recognition, therapeutic opportunities and understanding of the developmental, genetic and molecular origins of congenital heart diseases (CHDs)^3^. The etiology of the majority of CHDs, however, is poorly understood. Reasons are mainly due to their complexity because most of them are predicted to be multifactorial with elaborate genetic and environmental interactions in their etiology^3^. One severe form of CHD is Tetralogy of Fallot (TOF), representing the most common form of cyanotic CHDs. TOF is characterized by four features, namely a narrowing of the right outflow tract (pulmonary stenosis), a ventricular septal defect (VSD), an overriding aorta and right ventricular hypertrophy^4^. Recently, we showed an multigenic architecture of rare deleterious mutations in several genes, which discriminate isolated TOF cases from controls^5^. The affected genes are connected in an interaction network and play important roles in apoptosis and cell growth as well as in the assembly of the sarcomere and in the neural crest or secondary heart field. Moreover, all these genes function during heart development, the postnatal period and adulthood^5^. More recently, our findings have been confirmed by a larger cohort of patients^6^. Considering that TOF is suggested to be the result of altered proliferation, migration and differentiation of precardiac cells^7^, the lack of in vitro sources for human cardiomyocytes (CMs) has significantly impeded the investigation of this heart malformation.

The recent development of the induced pluripotent stem cell (iPSC) technology allows, for the first time, the study of human diseases using patient-specific cells in vitro^8^. Defined protocols for directed differentiation of human iPSCs to CMs represent the first step towards establishing cardiac development and disease models in vitro^9–12^. CMs generated from patient-specific iPSCs (ps-iPSCs) have been widely used as models for several cardiac diseases including channelopathies, cardiomyopathies and congenital heart defects, along with isolated CHD with complex genetics^13–15^. However, an in vitro model for TOF-specific CMs has not been described so far.

**Figure 1:**
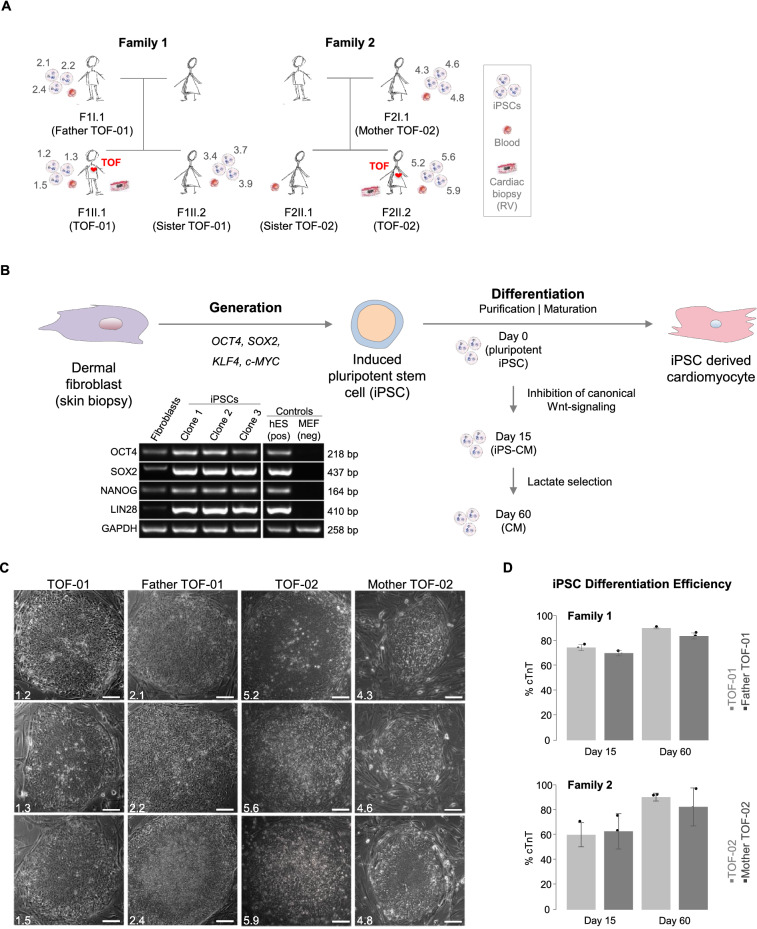
Generation of iPSCs from TOF patients and healthy relatives and their differentiation to cardiomyocytes. (**A**) Families with TOF patients (indicated by a red heart) and healthy family members. For each generated cell line, the individual clone identifier is provided (n = 3 for corresponding individual). Moreover, it is indicated whether genomic DNA was taken from blood for whole genome sequencing and/or from RV for targeted re-sequencing. (**B**) Generation of iPSCs and their differentiation to CMs. The gene expression of the pluripotency markers was also investigated at mRNA level by RT-PCR (here for iPSCs of TOF-01 exemplarily). The grouping of blots was cropped from different parts of the same gel, or from different gels. Uncropped blots are available in Supplementary Fig. S6. (**C**) Morphology of the generated iPSC colonies. Magnification 10×. Scale bar 100 µm. (**D**) Differentiation efficiency of CMs (each n = 3) at d15 and d60 between patients (light grey) and healthy relatives (dark grey). Cardiac differentiation efficiency is based on the percent of cTnT-positive CMs. CMs: cardiomyocytes; RV: right ventricle; TOF: Tetralogy of Fallot.

In this study, three ps-iPSC lines were generated from each of two well-characterized cases from our previously analyzed cohort of TOF patients^5^. In addition, 12 iPSC lines from healthy relatives serving as controls were generated (Fig. 1A). The respective cell lines were further successfully differentiated to CMs. The clonal mutational burden of the generated iPSCs was first analyzed by whole genome and transcriptome analysis. Moreover, there was shown to be potential disease-relevant differential gene expression between and across cardiomyocyte differentiation in TOF patients and healthy individuals. Overall, this study provides an in-depth understanding of cardiomyocyte differentiation and its transcriptional alterations in patients with TOF.

## Results

### Generation of iPSCs and differentiation to cardiomyocytes

Somatic cells were isolated from skin biopsies of two TOF patients and three healthy relatives of two unrelated families (Fig. 1A,B). For each individual, three independent iPSCs transduced from fibroblasts were established. The iPSCs are comparable to human embryonic stem cells (hESCs) and show the typical morphology of human iPSCs (Fig. 1C and Supplementary Figs. S1–5A,B). At mRNA level, the investigated pluripotency markers also indicated the pluripotent cell state of all iPSC lines (Fig. 1B and Supplementary Figs. S1–5C). Efficiency of the cardiac differentiation of the iPSCs was further evaluated by counting beating embryoid bodies. RT-PCR and immunocytochemistry experiments showed that the iPSCs were pluripotent and had the potential to differentiate into cells of all three germ layers (Supplementary Figs. S1–5D,E). Overall, the applied protocol resulted in functional, spontaneously beating heart muscle cells with features characteristic of physiological CMs. After 15 days of differentiation, cells were treated with lactate-supplemented medium for four days to enrich for a purer cardiomyocyte population (> 70%). The efficiency was validated by FACS-sorting against cTNT (cardiac troponin T). Forty days after induction of differentiation, iPSC-derived CMs showed a characteristic sarcomeric patterns, with no significant differences in differentiation efficiency between CMs of patients and controls in both families (Fig. 1D).

### Sequencing of iPSC samples

As iPSCs offer a platform to study genetic mutations associated with a disease, Sanger sequencing was performed on all rare deleterious single nucleotide variations (SNVs), which had been found by targeted re-sequencing in the right ventricular tissue of TOF-01 and TOF-02^5^. The sequence analysis of the iPSC lines confirmed that all mutations except one were present in the ps-iPSCs (Supplementary Tables S1–2). The confirmation of the mutations implied that the iPSCs would be suitable for studying the cellular and molecular phenotype. Next, whole genome sequencing (WGS) was performed on blood as well as of pooled iPSC clones (three clones at d0) from patients and their respective relatives to check for possible sporadic mutations occurring during iPSC generation either due to potential reprogramming-associated mechanisms or as a feature of patient-specific genome instabilty^16^. Individual clone-wise Sanger sequencing results were confirmed for known TOF gene mutations in the WGS samples of both blood and pooled iPSCs (Supplementary Tables S3-4). In addition, the differences between blood and iPSC samples were examined on a genome-wide scale based on local variations (SNVs as well as insertions and deletions, i.e., INDELs), copy number variations (CNVs) and structural variations (SVs).

**Figure 2:**
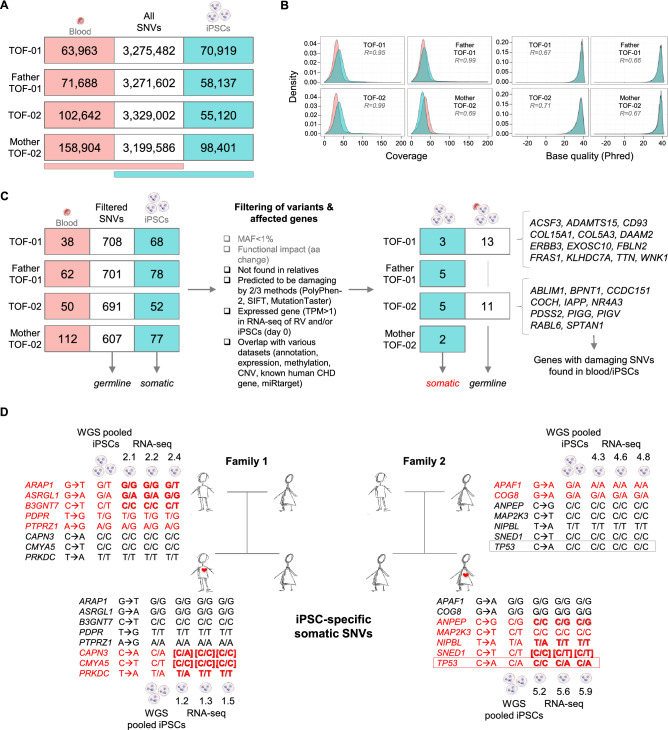
Identified candidate genes with somatic and/or germline single nucleotide variations in iPSCs of TOF patients and healthy relatives. (**A**) SNVs identified in blood and/or pooled iPSCs (n = 3). (**B**) Sequencing coverage and base quality for SNVs in blood (light red) or iPSCs only (cyan). (**C**) Filtering for disease candidate genes with somatic and germline SNVs. (**D**) Candidate genes with somatic SNVs. Whole genome sequencing was performed for pooled iPSCs and identified somatic SNVs were verified clone-wise (iPSC-specific) by whole transcriptome sequencing (RNA-seq). The individual somatic SNVs are marked in red. SNVs confirmed by Sanger sequencing are marked in bold. SNVs not verified by RNA-seq but confirmed by Sanger are marked by bold brackets. MAF: minor allele frequency; R: Spearman correlation; SNVs: single nucleotide variations; TPM: transcripts per kilobase million; WGS: whole genome sequencing.

In general, it was found that there was a very high overlap for all kinds of variations between the blood and iPSC samples (germline variants); only 1.6–3.0% SNVs in the pooled iPSC samples did not occur in the related blood samples (Fig. 2A and Supplementary Fig. S9). For these somatic variants, the sequencing coverage and base quality was compared as they may explain differences in the variant calling process. However, there was found to be a good correlation between blood and pooled iPSC samples over all individuals (Fig. 2B), indicating no sequencing-related bias. After initial variant calling, the raw variations were further filtered, which revealed almost no somatic and germline variations for healthy relatives (Fig. 2C and Supplementary Fig. S9).

Based on germline SNVs, 13 and 11 disease candidate genes were found in TOF-01 and TOF-02, respectively. In general, WGS resulted in different groups of candidate genes in both patients, which reflects the genetic heterogeneity of the disease. For TOF-01, the TOF-associated genes *ERBB3* (Erb-B2 receptor tyrosine kinase 3) and *TTN* (titin) were again among the candidate genes. Genes affected in TOF-01 were significantly enriched for collagen-related pathways as well as for the extracellular matrix organization pathway and related Gene Ontology (GO) terms. The affected genes in TOF-02 were significantly enriched for the glycosylphosphatidylinositol (GPI)-anchor-synthesis pathway and GO terms associated to metabolic processes. For INDELs or CNVs, no or no interesting hit, respectively, was found in the two TOF patients (Supplementary Fig. S9).

### Clonal mutational burden by reprogramming of iPSC

Focusing on disease-associated genes, which are potentially relevant for functional assays, further investigation was carried out for somatic and clonal variations that might interfere with disease modeling in iPSCs and derived CMs. For INDELs or larger variations, no relevant disease-related gene could be found. However, filtering for potential disease-related somatic SNVs revealed a few candidate genes (Fig. 2C). In addition to the results from WGS of the iPSC, clone-specific somatic mutations were identified in these genes based on the sequence read alignments of their individual RNA-seq data, which were also confirmed by Sanger sequencing (Fig. 2D and Supplementary Table S5).

**Figure 3:**
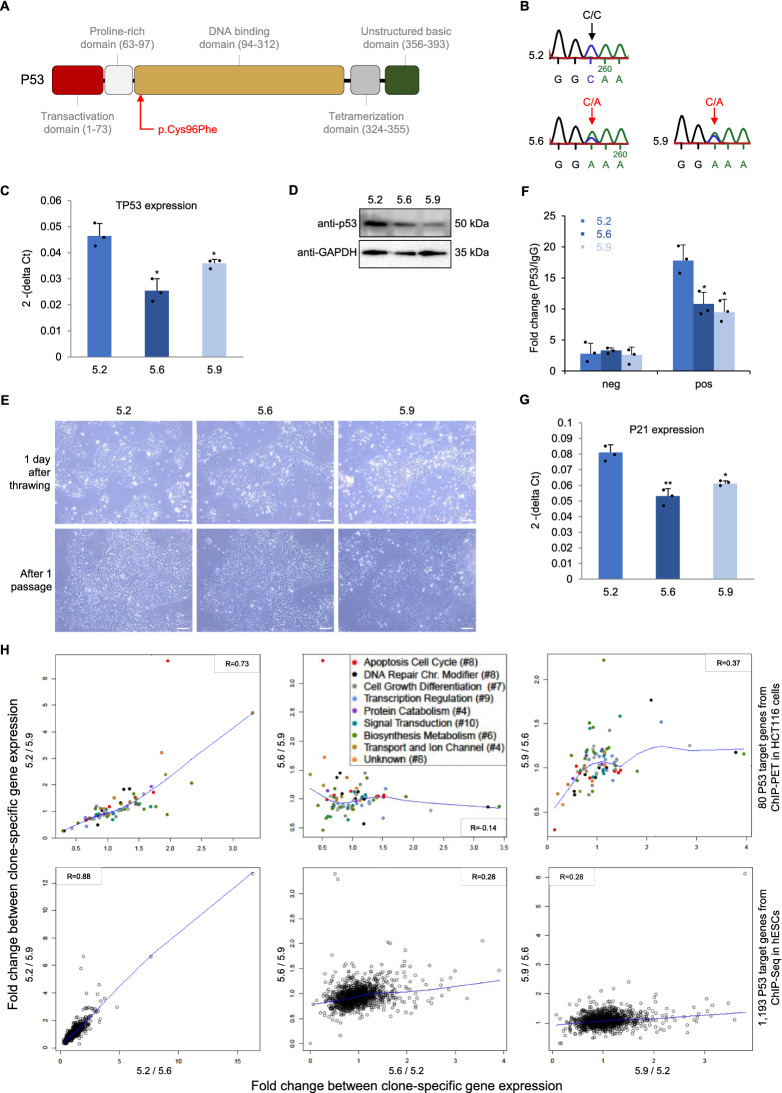
Somatic mutation in TP53 in iPSC clones of patient TOF-02. (**A**) Multifunctional domains of P53 (adapted from Robbins et al.^38^). The identified rare damaging SNV is located in the highly conserved DNA-binding core domain. (**B**) Sanger sequencing results. (**C**) Expression of TP53 in the individual iPSC clones of TOF-02 measured using quantitative real-time PCR. Expression was measured in triplicates and normalized to HPRT. (**D**) The protein level of P53 in the individual iPSC clones of TOF-02. GAPDH was used as the internal control. The grouping of gels/blots cropped from different parts of the same gel. Uncropped blots are available in Supplementary Fig. S10. (**E**) The comparison of TOF-02 iPSC morphology. The cells were maintained and propagated in E8 medium. Scale bar 240 µm. (**F**) The relative fold change of P53 enrichment between P21 positive region and negative region. (**G**) Expression of P21 in the individual iPSC clones of TOF-02 measured using quantitative real-time PCR. Expression was measured in triplicates (n = 3) and normalized to GAPDH. Significance was tested using a two-sided t-test. (**H**) Fold changes between clone-specific gene expression values of P53 target genes from ChIP-Seq in human embryonic stem cells (hESCs) and from ChIP-PET in HCT116 cells. The blue line indicates the locally-weighted polynomial regression (lowess fit). R: Pearson correlation; SNV: single nucleotide variation; TPM: transcripts per kilobase million. *P-value < 0.05.

Notably, a somatic mutation in *TP53*, encoding for the tumor protein P53, was found in two out of three iPSC clones of TOF-02 and not in the gDNA from blood of the patient. The identified mutation in *TP53* is located in the DNA binding domain of the protein (Fig. 3A), which was confirmed by Sanger sequencing (Fig. 3B). In general, iPSCs of TOF-02 have more variations compared to iPSCs of all other related or unrelated individuals (3.4 million vs. ~ 3.3 million SNVs and 1.49 million vs. ~ 1.47 million coding SNVs). Furthermore, expression of the gene *TP53* was observed to be significantly decreased (Fig. 3C) as well as its encoded protein P53 (Fig. 3D) in both affected clones compared to the unaffected one. Moreover, ps-iPS cell culture revealed that the affected clones seemed to be growing faster (Fig. 3E). The major transcriptional target of P53 in response to cellular stress is P21, which is a key component in cell cycle control and apoptosis^17^. As the DNA binding domain of P53 is affected by the mutation, P53 chromatin immunoprecipitation (ChIP) was performed at the promoter of P21 in all three iPSC clones of TOF-02. There was found to be significantly decreased binding of P53 at the promoter region of P21 in the affected clones (Fig. 3F). In line with this, the expression of P21 was also significantly decreased in both clones (Fig. 3G). Furthermore, the impact of the clone-specific mutation in P53 on a global scale was evaluated by correlating fold changes of clone-specific gene expression profiles of P53 target genes. The targets were obtained from available datasets on P53 ChIP-Seq in hESCs^18^ and ChIP-PET in primary cancer cells (HCT116)^19^. For both target gene sets, a strong correlation could only be found between the gene expression profiles of the two affected clones (Fig. 3H). This indicates that P53 target genes showed an altered gene expression profile at d0 in the affected clones. Moreover, these data clearly show that somatic mutations can alter the functional properties of both TOF-02-derived iPSCs and CMs. As this makes the cells inappropriate for studying the disease, we discarded TOF-02 for further analyses.

### Correlation of gene expression in iPSCs and CMs to healthy and TOF heart tissue

The expression profiles of iPSCs and CMs were correlated to corresponding gene expression in the right atrium and ventricle (RA and RV, respectively) as well as in the left atrium and ventricle (LA and LV, respectively) of healthy individuals, as well as to the RV expression profile of TOF patients (Supplementary Fig. S11A). In general, correlating expression profiles of iPSCs and CMs to heart tissue revealed nearly identical correlations for all origins. Thus, their profiles were combined for comparison to tissue profiles (Supplementary Fig. S11B). Overall, iPSCs correlated strongly with LA followed by LV, RA and finally, RV of healthy hearts. Furthermore, as expected, the correlation of expression profiles of iPSCs and derived CMs to tissues increased with the differentiation stage.

### Common gene expression pattern during cardiac differentiation

**Figure 4:**
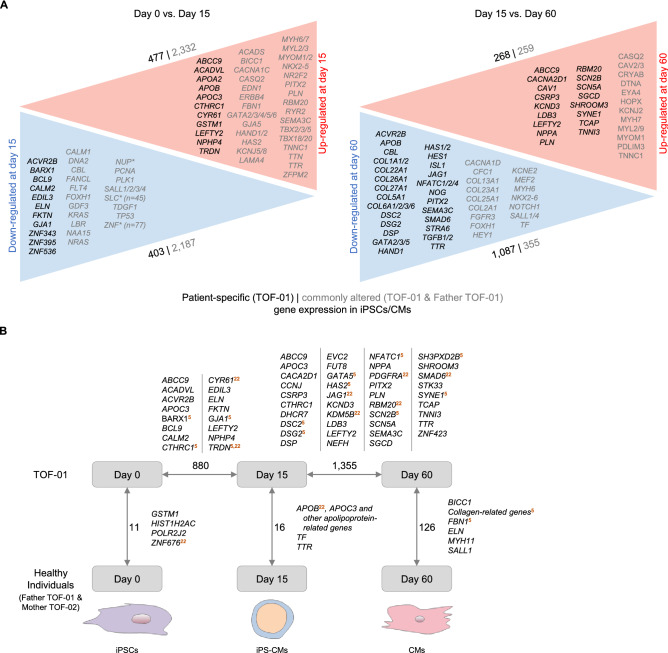
Differential gene expression between and across cardiac differentiation. (**A**) Differentially expressed genes between TOF-01 and Father TOF-01 for iPSCs versus CMs at d15 and CMs at d15 versus d60 (n = 3 for each individual and stage except CMs at d15 with n = 2). Patient-specific gene expression alterations at a specific state are shown in black and commonly altered gene expression (i.e., in both TOF-01 and Father TOF-01) are given in gray. The depicted genes are filtered for cardiac system-related genes. Multiple genes (> 2) of the same gene family are indicated by an asterisk. Differential gene expression is based on expressed autosomal protein-coding genes (adjusted *P*-value < 0.05, fold change > 2). (**B**) Overview of significantly differentially expressed genes (DEGs) in iPSCs and CMs of TOF-01 versus healthy individuals (Father TOF-01 and Mother TOF-02) and between differentiation stages of TOF-01 that are patient-specific (n = 3 for each individual and stage except CMs at d15 with n = 2 for TOF-01 and Father TOF-01). Cardiovascular-associated DEGs are listed and further marked if they overlap with findings based on genetic mutations, differential expression or methylation observed in our overall TOF cohort^5,22^.

RNAseq analysis of iPSCs (d0) and CMs (d15 and d60) provided insights into general expression profiles characterizing the differentiation process from iPSCs to cardiomyocytes across all samples (Fig. 4). Of note, this data analysis included normalization for cell composition. As expected, gene expression was altered from iPSCs to CMs at d15 and characterized by an up-regulation of genes associated with cardiac tissue development, cardiac ventricle morphogenesis and muscle cell differentiation. Up-regulated genes were significantly enriched for cardiac-, heart- or muscle-related GO terms and pathways.

### Patient-specific gene expression profiles during cardiac differentiation

In addition to study common expression profiles characterizing the differentiation process, we focused on patient-specific alterations during the differentiation of iPSCs to cardiomyocytes. These could highlight mechanism underlying a potential delay in differentiation or altered proliferation of cardiomyocytes eventually promoting the development of the structural defects observed in TOF.

Stage-specific alterations as well as dynamic changes across stages were analyzed. RNA-seq of iPSCs and CMs at d15 revealed 11 and 16 stage-specific genes, respectively, that were significantly differentially expressed between patient TOF-01 and healthy individuals (Fig. 4B). Among them are the cardiac-related gene *GSTM1* (Glutathione S-Transferase Mu 1) (Supplementary Fig. S12A) and genes known to be cardiac relevant like *TTR* (transthyretin; cardiac amyloidosis and hypertrophic cardiomyopathy) and members of the apolipoprotein family including *APOC3* (apolipoprotein C3; apolipoprotein C-III deficiency, Supplementary Fig. S12B,C). All of these genes are involved in various cellular transport activities with apolipoproteins being important components of many lipoproteins that are involved in cardiovascular disease^3,4,20,21^. As expected, based on the principle component analysis (Supplementary Fig. S13), the patient-specific expression profile at d60 became even more pronounced. A fourfold higher number of significantly down-regulated genes were observed in patient-CMs. These genes were significantly enriched for cardiovascular-associated GO terms and pathways, such as cardiac conduction or cardiovascular system development (Fig. 4 and Supplementary Fig. S14).

In addition to a stage-wise comparison of diseased and healthy expression profiles, we found patient-specific expression changes during the differentiation process across stages (Fig. 4). These included the significant down-regulation of several cardiac transcription factors (GATA factors, ISL1 (ISL LIM homeobox 1), HES1 (HES family BHLH transcription factor 1)), components of the NOTCH signaling cascade (e.g., JAG1 (jagged 1)), proteins relevant for cell–cell contacts (e.g., DSG2 (desmoglein 2)), and several genes coding for collagens. Beside this, genes handling ion transports were significantly up-regulated such as calcium or sodium channel genes (e.g., *CACNA2D1* (calcium voltage-gated channel auxiliary subunit alpha2delta 1), *SCN5A* (sodium voltage-gated channel alpha subunit 5)). Overall, specific differentially expressed genes (DEGs) showed a clear separation in expression between patient and healthy relative during cardiomyocyte differentiation. Importantly, comparison of the observed alterations was in line with previous findings based on genetic mutations, differential expression or methylation observed in our overall TOF cohort (Fig. 4B)^5,22^.

### Impact of variations on gene expression

**Figure 5:**
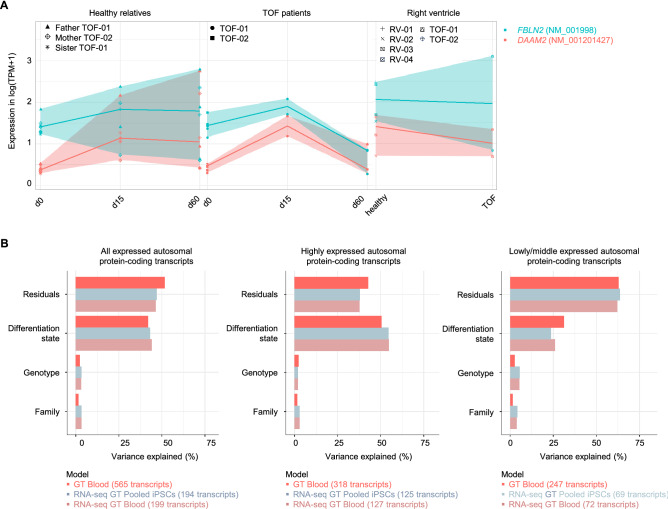
Differential gene expression and impact of variations. (**A**) Expression level of *FBLN2* and *DAAM2* in iPSCs and CMs of TOF patients and healthy individuals (n = 3 for each individual except CMs at d15 with n = 2 for TOF-01 and Father TOF-01; n = 0 for TOF-02 & sister TOF-01 at d15 & d60) as well as in the right ventricle of TOF patients and normal heart individuals. Both transcripts, which harbor mutations in TOF-01, were significantly differentially expressed between TOF-01 and Father TOF-01 in CMs at d60. (**B**) Impact of regulatory SNVs on transcriptional alterations. Linear mixed models were applied to all, highly and low/middle expressed autosomal protein-coding transcripts harboring I) SNVs called by WGS in the blood samples (‘GT blood’), II) SNVs called by WGS and confirmed by RNA-seq in the blood samples (‘RNAseq GT blood’) and III) SNVs called by WGS in the pooled iPSC samples and confirmed by RNA-seq of the individual clones (‘RNA-seq GT Pooled iPSCs’). GT: genotype; SNVs: single nucleotide variations.

In general, genetic variations of iPSCs and blood were not reflected in the expression profiles of the affected genes, as none of the DEGs in the two patients versus its healthy parents overlap with genes harboring germline or somatic SNVs in the patient’s blood and/or respective clones (Supplementary Fig. S15). This also holds true for CMs of d15 but not fully for the CMs at d60. For the latter, four DEGs were found in TOF-01 compared to Father TOF-01 with rare damaging germline SNVs in TOF-01. Out of these genes, only *DAAM2* (disheveled associated activator of morphogenesis 2) and *FBLN2* (fibulin 2) harbored a differentially expressed isoform affected by a mutation. Both isoforms were decreased in ps-CMs at d60 (Fig. 5A). The SNV in *FBLN2* was located in subdomain with a sevenfold decreased expression. The mutation in *DAAM2* was not located in a specific domain; however, the expression was also sevenfold decreased in TOF-01.

The global impact of regulatory SNVs on transcriptional variations was further investigated by applying linear mixed effect models with transcripts filtered for SNVs in proximal promoter regions that overlap with transcription factor binding sites (TFBS) (Fig. 5B). Overall, regulatory SNVs only had a minor effect on the transcriptional variation of the affected transcript, which is in line with the findings based on SNVs and DEGs at or between the different stages of cardiac differentiation. Considering highly expressed genes, most of the variance is explained by the differentiation state. This effect decreased for lower expressed transcripts. The family affiliation had only a small effect on transcriptional variation.

## Discussion

Induced pluripotent stem cells and derived cardiomyocytes are a valuable resource to model and to study diseases such as heart malformation^13,23^. In this study, ps-iPSCs were successfully generated and further differentiated into CMs to gain insights into TOF. Maturity and purity of CMs as well as their genetic integrity are methodological hurdles of iPSC applications. Simulations suggest there is a magnitude higher mutation intensity in iPSCs than the background mutation rate in culture due to induced reprogramming stress^24^. Although there are only small differences in the total numbers and types of variations among the different reprogramming methods, albeit most of the occurring variations are benign^25^. In general, differences in functional assays of iPSCs can be driven by genetic variability, either induced during reprogramming or as a feature of genomic instability underlying the disease^26^. Thus, to investigate somatic and clonal variations that might interfere with the disease modeling in vitro, we performed whole genome and transcriptome analyses from blood and/or iPSCs of the TOF patients and healthy relatives. Surprisingly, in two out of three clones of TOF-02, a somatic mutation was found in *TP53*, which encodes for the tumor protein P53. This protein acts as a tumor suppressor, meaning that it regulates cell division by preventing cells from growing and dividing too fast or in an uncontrolled way. Somatic mutations in the *TP53* gene are some of the most frequent alterations in human cancers^27^. In iPSCs, P53 is demonstrated to be required for maintaining genome integrity and it is expected to prevent accumulation of reprogramming-induced mutations^24,28^. In line with this, iPSCs of TOF-02 have more variations compared to iPSCs of all other related or unrelated individuals. Moreover, it was shown that in human ESCs the *TP53* mutant allelic fraction increased with passage number, which suggests that there is a selective advantage though P53 mutations^29^. Here, we discarded cells of TOF-02 for studying TOF; however, it clearly shows that supposedly disease-relevant somatic mutations can occur in iPSCs despite their low probability. Conclusions from the present study are limited by their sample size and it will be highly interesting to evaluate in further studies the probability of somatic mutations and genomic instability as an underlying feature of TOF.

During differentiation, an increasing number of DEGs was found between TOF-01 and healthy individuals. At the latest stage, several collagen-related genes were found to be differentially expressed. These genes harbor no mutations. However, it clearly shows that collagen-related genes are in general affected by genetic and/or transcriptional alterations in TOF. In addition to collagen-related genes, other potential disease-relevant ones were identified. For example, *BICC1* and *MYH11* were found to be down-regulated in CMs at d60 of TOF-01. The smooth muscle myosin MYH11 is usually absent from the heart but present in parts of the vasculature including the aorta^30^. The RNA-binding protein BICC1 acts as a negative regulator of the Wnt signaling pathway and might be involved in regulating gene expression during embryonic development^31^. It has been shown that rare deletions in TOF patients are significantly enriched for genes of the Wnt signaling pathways^4^. In addition to differential expression between two individuals at a certain differentiation stage, differential expression was observed across the different stages of maturation, specific for the particular individual or comparable with others. In general, genes which were specific differentially expressed between d15 and d0 in TOF-01 or Father TOF-01 were not enriched for cardiac system-related GO terms and/or pathways. In contrast, there was a clear difference in patient-specific DEGs compared to its healthy relative during cardiac differentiation. In fact, there was a higher number of down-regulated genes in CMs of TOF-01 at d60 versus d15 and moreover, these genes were enriched for cardiac system-related GO terms and pathways. Overall, the expression profiles of derived CMs differ increasingly between TOF-01 and Father TOF-01 and further segregate during cardiac differentiation, with clear differences regarding cardiac system-related expression at the later stages.

It was also checked whether genetic variations of iPSCs and derived CMs are reflected in the expression profiles of the affected genes. Globally, our approach is limited to SNVs in proximal promoter regions that overlap with TFBS. However, like others, we did not observe a correlation between genetic variations and expression alterations^5,32^. At d60, DEGs with potential disease-causing mutations in TOF-01, such as *FBLN2* and *DAAM2*, were found in TOF-01 CMs. *DAAM2* is co-required for myocardial maturation and sarcomere assembly^33^ and together with *DAAM1* (disheveled associated activator of morphogenesis 1), it nucleates actin and mediates Wnt-induced cytoskeletal changes^33^. *FBLN2* has an important role in cardiovascular development and smooth muscle cell migration und is also one of the candidate genes with deleterious variants in patients with down-syndrome-associated atrioventricular septal defects^34^. Furthermore, it is involved in the vascular endothelial growth factor-A (VEGF) signaling pathway, which is known to play a role in atrioventricular valvuloseptal morphogenesis^34^. VEGF and subsequent Notch signaling is important in cardiac outflow tract development and alterations can lead to abnormal development of myocardial structures^35^. Moreover, it is suggested that low expression VEGF haplotype increases the risk for TOF^36^.

In order to give insights into the molecular changes underlying the etiology of TOF, the establishment of ps-iPSCs and their differentiation to cardiomyocytes is assumed to be a valuable model. The results of this study are merely based on three clones each from one patient and healthy relatives; however, they show clear transcriptional differences in CMs of the patient, which needs to be verified by follow-up studies with more cases. Moreover, modeling TOF is challenging as it is characterized by several malformations at the organ level occurring at once. Heart organoids or other three-dimensional models might therefore be better choices to investigate severe heart malformations. Nevertheless, our results based on monolayer CMs cultures, which model the muscular rather than the structural phenotype, are promising for further investigations. Moreover, they contribute to the further understanding of this disease, for which an in vitro model has not been described so far, neither as 2D or 3D model. Lastly, we used healthy relatives instead of isogenic lines as controls for this study. The rationale behind is that TOF is a complex oligogenic disorder with unknown exact genetic causes^3^. Moreover, isogenic lines introduce variability due to gene editing off-target events and stress caused by additional rounds of culture selection, whereas cell lines from patients and relatives carrying the same genetic background more accurately mirrors the real situation^37^.

In summary, iPSC technology was employed in the present study to model and to study TOF-specific cardiomyocytes for the first time in vitro. Indeed, clear transcriptional differences between CMs of the patient and its healthy relative were observed, in particular at the later stages of cardiac differentiation. Our findings provide novelty into the transcriptional landscape of TOF and potentially improve our understanding of right ventricular heart failure, which is the major long-term phenotype of TOF patients.

## Methods

### Study participants and ethical statement

Skin biopsies for iPSC generation were obtained from three members of family 1 (from the patient TOF-01 and from his unaffected father and sister) and two members of family 2 (from the patient TOF-02 and her unaffected mother) (Fig. 1A). Further, blood samples for DNA analysis were taken from the three members of family 1 as well as family 2 as described previously^5^. Cardiac biopsies from the right ventricle were taken from the two TOF patients as described previously^5^. In addition, biopsies were taken from eight other TOF patients and 16 healthy individuals. The local institutional review board of the Charité – Universitätsmedizin Berlin approved the study and informed consent was obtained from all participants or guardians. The study protocol conforms to the ethical guidelines of the 1975 Declaration of Helsinki.

### Generation of iPSCs and differentiation into cardiomyocytes

iPSCs were generated from skin biopsies of two TOF patients and three healthy relatives using STEMCCA system, containing all four reprogramming factors OCT4, SOX2, KLF4, and c-MYC. For each individual, three iPSC clones were generated^12^. The cardiac differentiation was carried out by manipulating Wnt-signaling, applying small molecules under fully defined conditions. CMs were selected after day 15 using lactate-supplemented medium for four days.

### Whole genome and transcriptome sequencing

Genomic DNA (gDNA) from blood of patients and healthy relatives was extracted using QIAamp DNA Blood Midi Kit (Qiagen). gDNA from three iPSC clones was pooled for each individual before library preparation and sequencing. Each sample was prepared according to the Illumina TruSeq DNA sample preparation guide to obtain a final library of 300–400 bp average insert size. Whole genome sequencing (WGS) of the blood and pooled iPSCs samples was performed by Macrogen using Illumina HiSeq X (2 × 150 bp paired-end sequencing, 30 × coverage). On average, sequencing resulted in 77 million reads per sample.

Total RNA was isolated and purified to get poly(A) RNA. The RNA libraries were prepared with the ScriptSeq v2 RNA-Seq Library Preparation Kit (Illumina). Paired-end sequencing was performed on either HiSeq 2,500 (2 × 51 bp) or NextSeq (2 × 76 bp) (Illumina). All kits were applied according to the manufacturer's instructions. On average, mRNA sequencing (RNA-seq) resulted in 65 million reads per sample.

### Statistics

General bioinformatics and statistical analyses were conducted using R (including Bioconductor packages) and Perl.

### Data access

RNA-seq data of iPSCs and derived CMs are available from the Gene Expression Omnibus (GEO) repository at NCBI (accession number GSE111230).

## Supplementary information

Supplementary file1 (PDF 2907 kb)

Supplementary file2 (XLSX 287 kb)

Supplementary file3 (XLSX 322 kb)

Supplementary file4 (XLSX 12 kb)

Supplementary file5 (XLSX 12 kb)

Supplementary file6 (XLSX 162 kb)

Supplementary file7 (XLSX 10 kb)

Supplementary file8 (XLSX 10 kb)

Supplementary file9 (XLSX 11 kb)

Supplementary file10 (XLSX 11 kb)

## References

[1] Marelli AJ (2014). Lifetime prevalence of congenital heart disease in the general population from 2000 to 2010. Circulation.

[2] van der Linde D (2011). Birth prevalence of congenital heart disease worldwide: a systematic review and meta-analysis. J. Am. Coll. Cardiol..

[3] Rickert-Sperling S, Kelly RG, Driscoll DJ (2016). Congenital Heart Diseases: The Broken Heart. Clinical Features, Human Genetics and Molecular Pathways.

[4] Lahm H (2015). Tetralogy of Fallot and hypoplastic left heart syndrome—complex clinical phenotypes meet complex genetic networks. Curr. Genomics.

[5] Grunert M (2014). Rare and private variations in neural crest, apoptosis and sarcomere genes define the polygenic background of isolated Tetralogy of Fallot. Hum. Mol. Genet..

[6] Page DJ, Miossec MJ, Williams SG (2018). Whole exome sequencing reveals the major genetic contributors to non-syndromic Tetralogy of Fallot. Circ. Res..

[7] Di Felice V, Zummo G (2009). Tetralogy of fallot as a model to study cardiac progenitor cell migration and differentiation during heart development. Trends Cardiovasc. Med..

[8] Takahashi K (2007). Induction of pluripotent stem cells from adult human fibroblasts by defined factors. Cell.

[9] Burridge PW, Matsa E, Shukla P, Lin ZC, Churko JM (2014). Chemically defined generation of human cardiomyocytes. Nat. Methods.

[10] Lian X, Zhang J, Azarin SM, Zhu K, Hazeltine LB (2013). Directed cardiomyocyte differentiation from human pluripotent stem cells by modulating Wnt/β-catenin signaling under fully defined conditions. Nat. Protoc..

[11] Kattman SJ (2011). Stage-specific optimization of activin/nodal and BMP signaling promotes cardiac differentiation of mouse and human pluripotent stem cell lines. Cell Stem Cell.

[12] Cyganek L (2018). Deep phenotyping of human induced pluripotent stem cell-derived atrial and ventricular cardiomyocytes. JCI Insight.

[13] Doyle MJ (2015). Human induced pluripotent stem cell-derived cardiomyocytes as a model for heart development and congenital heart disease. Stem Cell Rev..

[14] Wang G (2014). Modeling the mitochondrial cardiomyopathy of Barth syndrome with induced pluripotent stem cell and heart-on-chip technologies. Nat. Med..

[15] Itzhaki I (2011). Modelling the long QT syndrome with induced pluripotent stem cells. Nature.

[16] Sugiura M (2014). Induced pluripotent stem cell generation-associated point mutations arise during the initial stages of the conversion of these cells. Stem Cell Rep..

[17] Hill R, Bodzak E, Blough MD, Lee PWK (2008). p53 binding to the p21 promoter is dependent on the nature of DNA damage. Cell Cycle.

[18] Akdemir KC (2013). Genome-wide profiling reveals stimulus-specific functions of p53 during differentiation and DNA damage of human embryonic stem cells. Nucleic Acids Res..

[19] Wei C-L (2006). A global map of p53 transcription-factor binding sites in the human genome. Cell.

[20] Rodemoyer A (2014). A tissue-specific gene expression template portrays heart development and pathology. Hum. Genomics..

[21] Rhee E-J, Byrne CD, Sung K-C (2017). The HDL cholesterol/apolipoprotein A-I ratio: an indicator of cardiovascular disease. Curr. Opin. Endocrinol. Diabetes Obes..

[22] Grunert M (2016). Comparative DNA methylation and gene expression analysis identifies novel genes for structural congenital heart diseases. Cardiovasc. Res..

[23] Matsa E, Burridge PW, Wu JC (2014). Human stem cells for modeling heart disease and for drug discovery. Sci. Transl. Med..

[24] Ji J (2011). Elevated coding mutation rate during the reprogramming of human somatic cells into induced pluripotent stem cells. Stem Cells.

[25] Bhutani K (2016). Whole-genome mutational burden analysis of three pluripotency induction methods. Nat. Commun..

[26] Burrows CK (2016). Genetic variation, not cell type of origin, underlies the majority of identifiable regulatory differences in iPSCs. PLoS Genet..

[27] Olivier M, Hollstein M, Hainaut P (2010). TP53 mutations in human cancers: origins, consequences, and clinical use. Cold Spring Harb. Perspect. Biol..

[28] Marión RM (2009). A p53-mediated DNA damage response limits reprogramming to ensure iPS cell genomic integrity. Nature.

[29] Merkle FT (2017). Human pluripotent stem cells recurrently acquire and expand dominant negative P53 mutations. Nature.

[30] England J, Loughna S (2012). Heavy and Light Roles: Myosin in the Morphogenesis of the Heart, Vol. 70.

[31] Kraus MR-C (2011). Two mutations in human BICC1 resulting in Wnt pathway hyperactivity associated with cystic renal dysplasia. Hum. Mutat..

[32] Postma AV, Bezzina CR, Christoffels VM (2015). Genetics of congenital heart disease: the contribution of the noncoding regulatory genome. J. Hum. Genet..

[33] Ajima R (2015). DAAM1 and DAAM2 are co-required for myocardial maturation and sarcomere assembly. Dev. Biol..

[34] Ackerman C (2012). An excess of deleterious variants in VEGF-A pathway genes in Down-syndrome-associated atrioventricular septal defects. Am. J. Hum. Genet..

[35] van den Akker NMS (2007). Tetralogy of fallot and alterations in vascular endothelial growth factor-A signaling and notch signaling in mouse embryos solely expressing the VEGF120 isoform. Circ. Res..

[36] Lambrechts D (2005). Low expression VEGF haplotype increases the risk for tetralogy of Fallot: a family based association study. J. Med. Genet..

[37] Gacita AM, Puckelwartz MJ, McNally EM (2018). Modeling human dilated cardiomyopathy using humans. JACC Basic Transl. Sci..

[38] Robbins D, Zhao Y (2012). Oxidative stress induced by MnSOD-p53 interaction: pro-or anti-tumorigenic?. J. Signal Transduct..

