# Supplementary material for: Induced pluripotent stem cells of patients with Tetralogy of Fallot reveal transcriptional alterations in cardiomyocyte differentiation
Source: Sci Rep. 2020 Jul 2;10:10921. doi: 10.1038/s41598-020-67872-z (PMC7331606; doi:10.1038/s41598-020-67872-z)
Supplement: Supplementary file 1 — Supplementary file1 (PDF 2907 kb) [file 41598_2020_67872_MOESM1_ESM.pdf]

Induced pluripotent stem cells of patients with Tetralogy of Fallot reveal transcriptional alterations in cardiomyocyte differentiation

Marcel Grunert, Sandra Appelt, Sophia Schönhals, Kerstin Mika, Huanhuan Cui, Ashley Cooper, Lukas Cyganek, Kaomei Guan & Silke R. Sperling

Supplementary Information

Supplementary Methods

Generation and culture of iPSCs. iPSCs were generated from skin biopsies of two TOF patients and three healthy relatives using STEMCCA system, containing all four reprogramming factors OCT4, SOX2, KLF4, and c-MYC in a single stem cell cassette (pHAGE2-EF1aFull-hOct4-F2A-hKlf4 IRES hSox2-P2A hcMyc-C-loxP). For each individual, three independent iPSCs were established to reduce the phenotypic variability between the cell lines (Supplementary Table S6)[1]. The cells were kept in an incubator at 37°C with 5% CO2 in coated culture dishes. All undifferentiated cells were treated with E8 full medium (Thermo Fisher Scientific). The media was changed every 24 h and cells were split as needed with 90% confluence. For splitting, the cells were washed once with DMEM/F12 (Thermo Fisher Scientific) and then treated with collagenase IV (Thermo Fisher Scientific) for 2 min at 37°C. The collagenase was washed off once with DMEM/F12 and the cells were scraped off the culture dish in 2 ml hES medium (Thermo Fisher Scientific). Using a glass pipette, the size of cell clumps was carefully reduced and the cells were seeded onto a new culture dish.

The iPSCs were further analyzed to verify their pluripotency. They were all comparable to human embryonic stem (hES) cells and showed the typical morphology of human iPSCs. The cells grew in colonies with a high density, flat and round shapes as well as distinct colony edges. Furthermore, the colonies were positive for alkaline phosphatase activity staining (Supplementary Fig. S1-5A). Immunohistologically, all iPSCs were comparable to hES cells for staining with human stem cell markers, including OCT4, SOX2, NANOG, LIN28, SSEA-4, and TRA-1-60 (Supplementary Fig. S1-5B). On mRNA level, the investigated pluripotency markers also indicated the pluripotent cell state of all iPSC lines (Supplementary Fig. S1-5C). Even though the expression of NANOG was also present in the TOF fibroblasts, the difference in the expression level was higher in the iPSC lines. Furthermore, no or only very weak expression of the key regulator SOX2, OCT4 and the miRNA regulator LIN28 could be observed in the fibroblasts. In contrast, the expression level of those pluripotency markers in the iPSCs was comparable to the hES cell line. Given the age of the cell lines (in culture for over four months and around 20 passages), the expressed pluripotency markers are endogenous.

Differentiation into cardiomyocytes. The cardiac differentiation was carried out by manipulating Wnt-signaling, applying small molecules under fully defined conditions. Briefly, iPSCs were cultured on Geltrex (Thermo Fisher Scientific) coated 6-well plates in E8 medium (Thermo Fisher Scientific) for 5-6 days. To initiate differentiation, iPSCs were seeded on Geltrex coated plate at a density of 2 million in E8 medium supplemented with Pro Survival compound (Merck). The medium was changed daily, and after 2 days when the monolayer of cells reached 100% confluence, the medium was changed to RPMI supplemented with B27

minus insulin (Thermo Fisher Scientific) containing the GSK3-inhibitor XVI (CHIR99021; Merck). This day was labeled as day 0 of differentiation (pluripotent state). On day 1 of differentiation, the medium was changed to RPMI supplemented with B27 minus insulin. On day 3, medium was changed to RPMI supplemented with B27 minus insulin containing the Wnt II antagonist IWP-2 (Merck). On day 5, the medium was changed to RPMI supplemented with B27 minus insulin. Starting at day 7, the medium was changed to RPMI supplemented with B27 complete supplement and refreshed every three days. In order to get a cardiac differentiation efficiency of 70% or higher[2], cardiomyocytes were selected after day 15 using lactate-supplemented medium for 4 days (d19-d23; Supplementary Fig. S16). Lactate selection is efficiently used for energy metabolisms while non- cardiomyocytes undergo apoptosis in glucose depleted media[3].

Cardiac differentiation efficiency of the iPSCs was evaluated by counting beating embryoid bodies. Reverse transcription PCR analyzes showed that important genes for the development of the endoderm (alpha fetoprotein and albumin), mesoderm (major histocompatibility complex, cardiac troponin type 2 and ventricular myosin light chain-2) and ectoderm (tyrosine hydroxylase and synapsin I) were expressed in their expected patterns (Supplementary Fig. S1-5D). Moreover, immunocytochemistry showed the specific endodermal marker AFP, the mesodermal marker SMA (SM-α-actin) and the ectoderm-specific marker ß- tubulin in the differentiated cells (Supplementary Fig. S1-5E). After subcutaneous injection of the iPS cells in immunodeficient mice that did not have B, T, and NK cells (RAG2-/-γc-/-), all cell lines produced teratoma, with derivatives of all three germ layers being observed. In summary, the experiments indicated that the iPSCs were pluripotent with the potential to differentiate in vitro and in vivo into cells of all three germ layers.

Cardiac differentiation of the generated iPSCs (day 0) into the mesodermal (day 1) and the cardiac progenitor stage (day 5 and day 8) as well as the functional (day 15) and mature cardiomyocytes (day 60) was performed under chemically defined conditions in feeder-free culture[4]. Via the inhibition of GSK-3 (glycogen synthase kinase 3), iPSCs were induced to differentiate towards the mesodermal lineage while temporal modulation of canonical Wnt-signaling mediated by porcupine inhibitors directed mesodermal cell fate towards the cardiac lineage three days after induction of differentiation[4]. Upon differentiation, iPSCs developed into spontaneously contracting cardiomyocytes while running through differentiation stages of the mesodermal and the cardiac progenitors. The successful establishment of a cardiac differentiation protocol was confirmed by semiquantitative RT-PCR. Moreover, flow cytometry and immunocytochemistry were performed for mesodermal and cardiac marker.

Alkaline phosphatase staining. The alkaline phosphatase (ALP) staining was performed with the AP-Kit (Sigma). The medium of a moderately grown plate was discarded and the cells

were washed once with 1x PBS. For fixation of the cells, fixation solution (5 ml citrate solution, 13 ml acetone, 1.6 ml 37% formaldehyde) was added to the cells for 30 sec. Subsequently, the cells were washed with H_2O for about 1 min. For the staining solution 0.3 ml sodium nitrate was mixed with 0.3 ml FBB-Alk and kept for 2 min at RT before 13.5 ml H_2O was added. Just before applying it onto the cells, 0.3 ml Naphthol AS-B (Sigma) was added. The staining solution was kept on the cells for 15 min at RT and then washed away with H_2O. The cells were left to dry at RT.

Immunocytochemistry (pluripotency markers). The cells were fixed with either 4% paraformaldehyde (PFA) or methanol and acetone. For the 4% PFA fixation, the medium of the cells was discarded and the culture dish was washed 2-3x with PBS. 4 ml PFA was added and the cells were incubated for 20 min at RT. The PFA was discarded and the cells are washed 4x with PBS until the PFA was completely washed off. 3-5 ml 1% BSA was then added and the culture dish was sealed with parafilm. Until use, the cells were stored at 4 °C. For the methanol and acetone fixation, the medium of the cells was discarded and the cells were washed 2-3x with PBS. Then 4 ml ice-cold methanol-acetone solution (70% methanol, 30% acetone) was added and the cells were incubated at -20 °C for 10 min. The methanol-acetone was discarded and the cells were washed 4x with PBS. 3-5 ml 1% BSA was added and the culture dish was sealed with parafilm. The cells were stored at 4°C until use.

The cover slips with the fixed cells were placed in a wet chamber. 0.1% Triton-X was added to the cells if SOX2, OCT4, LIN28 or NANOG were to be stained. These cells were then washed 3x with PBS. The primary antibodies used are listed in Supplementary Table S7 and the cells were incubated with the primary antibody at 37 °C for 1 h. After that, the cover slips were washed 3x with PBS and incubated with the secondary antibody at 37 °C for 1 h. After washing 3x with PBS the cells were incubated with DAPI at RT for 10 min in the dark. The cover slips were then washed 3x with PBS and once with H_2O. For mounting, the cover slips were placed upside down on Vectashield and sealed with nail polish.

PCR of pluripotency markers. The RNA isolation from fibroblasts and iPS cells was performed using the Promega RNA isolation kit. Fibroblast pellets were lysed in 500 µl lysis buffer (10 ml RNA-Buffer, 200 µl β-mercaptoethanol) and iTOF pellets in 700 µl lysis buffer. The same amount of 95% ethanol was added, mixed and transferred into a filter tube. The samples were centrifuged at 12,000 rpm for 1 min and the liquid was discarded. The RNA and DNA were now present on the filter. 600 µl RNA wash solution was added onto the filter and the samples were centrifuged for 1 min at 12,000 rpm. The liquid was discarded. 50 µl DNase Incubation Mix (40 µl Yellow Core Buffer, 5 µl MnCl2 (0.09M), 5 µl DNase I) was given to each sample and incubated for 15 min at RT. 200 µl DNase Stop Solution was then added and

centrifuged for 1 min at 12,000 rpm. After adding 600 µl RNA Wash Solution the samples were centrifuged at 12,000 rpm for 1 min and the liquid was discarded. Another 250 µl RNA Wash Solution was added, and the samples were centrifuged at 12,000 rpm for 2 min. The liquid was discarded and the filter placed in a new RNA-free tube. 100 µl RNase- free water (DEPC-H2O) was added onto the filter and the samples were centrifuged at 1,2000 rpm for 1 min.

The RNA concentration of the eluate was measured with a photometer and the samples stored at -20 °C until use. The prepared RNA samples were converted into cDNA by RT. Therefore, 200 ng RNA, 2 µl 10x PCR Buffer II, 4 µl MgCl2 (25 mM), 0.8 ml dNTPs (100 mM), 1 µl RNase Inhibitor, 1 µl Oligo (dt) 16 and 1 µl MuLV reverse Transcriptase were added into a PCR tube. Depending on the volume of the RNA the sample was volumized to 20 µl with DEPC- H2O.

The prepared cDNA was used for proving the expression of pluripotency markers in the iTOF cells. These markers included the genes NANOG, OCT4, LIN28, SOX2, and KLF4. The housekeeping gene GAPDH was used as a control. For the PCRs 2 µl cDNA, 30.1 µl DEPC- H_2O, 10 µl 5x GoTaq Buffer I, 3.2 µ dNTPs (10 mM), 2 µl forward primer, 2 µl reverse primer, 0.2 µl GoTaq Polymerase and 0.5 µl DMSO was added into a PCR tube. The PCR products were run on a 1.5% agarose gel by electrophoresis.

Validation of genomic variations by Sanger sequencing. PCR reactions were carried out using gDNA templates and standard protocols. The primers are listed in Supplementary Table S1-2. Sanger sequencing was carried out with Eurofins Genomics.

Whole genome sequencing (WGS). Genomic DNA (gDNA) from blood of patients and healthy relatives was extracted using QIAamp DNA Blood Midi Kit (Qiagen). The isolation of gDNA from ps-iPSCs and iPSCs of one healthy parent from each family was carried out using Quick-DNA™ Miniprep Kit (Zymo Research). Furthermore, gDNA from three iPSC clones was pooled for each individual before library preparation and sequencing. Each sample (in total 10) was prepared according to the Illumina TruSeq DNA sample preparation guide to obtain a final library of 300-400 bp average insert size. WGS of the blood and pooled iPSCs samples was performed by Macrogen using Illumina HiSeq X (2x150bp paired-end sequencing, 30x coverage). On average, sequencing resulted in approximately 77 million reads per sample (Supplementary Fig. S17 and Table S8). The quality of the sequencing samples was checked using FASTQC version 0.11.3 (http://www.bioinformatics.babraham.ac.uk/projects/fastqc/). All samples passed sequence quality and had an average duplication rate of 10.4%. After initial quality check (Supplementary Fig. S17), the reads were mapped to the human reference genome (GRCh38.p3/hg38) using Bowtie2 with the 'very-sensitive' parameter setting, which is thought to be more sensitive and more accurate[5]. On average, 97.9% of the reads per sample

could be mapped to unique genomic locations and out of these 82.2% could be mapped uniquely. Duplicate reads were located and tagged with Picard tools version 1.140 (http://broadinstitute.github.io/picard).

Local variation calling (single nucleotide variations; SNVs as well as insertions and deletions; INDELs) was performed with GATK version 3.4-46 according to best practice pipelines including INDEL realignment, base recalibration and genotyping using HaplotypeCaller[6]. Post-processing steps included GATKs Variant Quality Score Recalibration (VQSR)[7] and variant annotation using dbSNP build 144. On average, 3.3 million SNVs and 0.8 million INDELs were called per sample (Supplementary Fig. S17). Variants that passed GATK default quality filters were annotated using snpEff version 4.2[8]. After annotation, local variations were filtered for rare ones with functional impact. A rare variant was defined by having a minor allele frequency of 1% in the 1000 Genomes Project (Phase 1 release v3) or in the NHLBI GO Exome Sequencing Project (4,300 European-American unrelated individuals). A variation was further categorized as having a functional impact, if it was annotated with one or more of the following criteria: frameshift, non-synonymous coding, change of splice site, loss or gain from stop/start codon. In addition, variations were predicted to be damaging in at least two out of three tools (PolyPhen-2[9], SIFT[10] and MutationTaster2[11]). Copy number variations (CNVs) were called using Control-FREEC (version 8.7 with default parameters), which applied a read depth-based approach normalized by GC content[12]. Structural variations (SVs) were called using Manta (version 0.29.2 with default parameters)[13]. Called CNVs and SVs were further overlapped with genomic features obtained from GENCODE v22[14].

Filtering for disease-relevant genes by overlapping with various datasets. Candidate genes with genomic variations were overlapped with various datasets to filter for known or possible disease-relevant genes. This includes I) heart- and muscle relevant genes (865 genes based on several resources[15]); II) cardiovascular-associated genes (list of 4,275 genes created by the Cardiovascular Gene Annotation Initiative in collaboration with EMBL-EBI); III) genes which are differentially expressed in CHD patients (mainly TOF, VSD, ASD, HLHS, TGA-PS)[15-20]; IV) genes which are differentially methylated in the promoter, gene body or related enhancer of CHD patients (mainly TOF, VSD, LS-CHD)[21-25]; V) genes which overlap copy number variations associated with CHD (including TOF, LS-CHD, HLHS and conotruncal defects)[26-41]; VI) known human CHD genes[37,42,43]; and finally VII) genes which are differentially expressed and targeted by differentially expressed microRNAs in CHD patients (TOF and HLHS patients only)[44,45].

Post-processing of P53 ChIP-Seq and CHIP-PET data. Chromatin immunoprecipitation (ChIP) followed by paired-end tag sequencing (ChIP-PET) of P53 in primary cancer cells (HCT116) resulted in 542 binding loci with high confidence, with 122 previously known or novel P53 target genes[46]. These targets were further filtered to be expressed (TPM>1) in at least one of three iPSC clones (day 0) of TOF-02, which resulted in 80 genes. ChIP followed by sequencing (ChIP-Seq) of P53 in H9 human embryonic stem cells (hESCs; H9-derived) resulted in 4,526 binding sites[47]. The ChIP peak positions were overlapped (at least 1 bp) with promoter regions (2,000 bp upstream and 500 bp downstream from transcriptional start site) of protein-coding genes from RefSeq. Genes with peaks in their promoter region were further filtered to be expressed (TPM>1) in at least one of three iPSC clones (day 0) of TOF-02, which finally resulted in 1,193 target genes.

RNA sequencing. Total RNA was isolated using Direct-zol RNA Kits (Zymo Reasearch) and purified with Dynabeads Oligo(dT)$_{25}$ (Thermo Fisher Scientific) to get poly(A) RNA. The RNA libraries were prepared with the ScriptSeq v2 RNA-Seq Library Preparation Kit (Illumina). The libraries were evaluated with the Qubit dsDNA HS Assay Kit (Thermo Fisher Scientific) and the Agilent High Sensitivity DNA Kit (Agilent Technologies). Paired-end sequencing was performed on either HiSeq 2500 (2x51 bp) or NextSeq (2x76 bp) device (Illumina). For the latter, the NextSeq 500/550 v2 kits (Illumina) were used. All kits were applied according to the manufacturer's instructions. Overall, RNA sequencing was performed for 31 samples of 5 individuals for the following three differentiation stages: pluripotent iPSC (day 0), iPSC-CM (day 15) and mature CM (day 60). Sequencing was separated into two runs, all day 0 samples and three day 15 samples were sequenced with Illumina's HiSeq 2500. The remaining samples were sequenced using Illumina's NextSeq. Sequencing quality was estimated using FASTQC. All samples passed sequence quality and had an average duplication rate of 41%. On average, mRNA sequencing resulted in 65 million reads over all samples (Supplementary Fig. S18 and Table S9). Reads were mapped to the human reference genome (GRCh38/hg38) using STAR v2.4.2a with basic 2-pass mapping[48] and the GENCODE gene/transcript annotation set v22[14]. On average 55.5 million reads could be mapped to unique genomic locations (Supplementary Fig. S18 and Table S9). Gene- and transcript-based expression values, including read count and transcripts per million (TPM), were calculated using RSEM v1.2.31 with GENCODE v22 annotations[49] for a strand-specific protocol. In total, there were 16,903 protein-coding genes and 52,429 protein-coding transcripts with TPM > 1 in at least one sample.

Correlation to healthy and TOF heart tissue. Total RNA library preparation was performed for four samples each of right ventricle, right atrium, left ventricle and left atrium of healthy hearts as well as for ten samples of the right ventricle of TOF patients (including TOF-01 and TOF-02). Paired-end sequencing was performed on Illumina HiSeq 4000 (2x76bp).

Reads were mapped to the human reference genome (GRCh38) and transcriptome (Gencode v22 annotations) using Star v2.4.2a. Gene and transcript expression levels were calculated as TPM (and FPKM) using RSEM v1.2.31 with Gencode v22 annotations. For the correlation, expressed (TPM > 1 in at least one sample) protein-coding genes were considered (19,216 genes) and for each state the median TPM was used.

Identification of differentially expressed genes. To determine specific and commonly differentially expressed genes and their expression patterns, genes differentially expressed between day 0, day 15 and day 60 (comparison of 3 versus 3 cell lines) of patients and healthy relatives or individuals () were identified using EdgeR v3.16.5[50] based on expressed autosomal protein-coding genes (TPM>1 in at least on cell line in at least one group). Genes were defined as significantly differentially expressed if the adjusted p-value was below 0.05 using the Benjamini-Hochberg correction for controlling the false discovery rate (FDR) and moreover, if the fold change was greater or equal to two.

DEGs between the differentiation stages (i.e., between different stages of the same individual or between lines of the same stage) might be the results of inherent line to line differences in differentiation efficiency/diversity of cell types. This issue was solved indirectly by searching for individual/patient-specific DEGs based on similar differentiation efficiencies between the three cell lines of individuals at stage day 15 and day 60 (i.e., day 15 TOF-01 vs. Father TOF-01 p=0.1; day 15 TOF-01 vs. Mother TOF-02 p=0.4; day 60 TOF-01 vs. Father TOF-01 p=0.1; day 15 TOF-01 vs. Mother TOF-02 p=0.7; p-values based on *Mann–Whitney U* test of the percent of cTNT-positive cardiomyocytes; see also Fig.1D). Moreover, differentiation efficiencies between the different stages (day 15 and day 60) within cell lines of the same individual are also not significantly different (i.e., day 15 TOF-01 vs. day 60 TOF-01 p=0.1 with a mean difference of 15.6% cTnT as well as day 15 Father TOF-01 vs. day 60 Father TOF-01 p=0.1 with a mean difference of 13.7% cTnT). While searching for patient-specific DEGs, all DEGs in TOF-01 were discarded that are also differentially expressed between the same differentiation stages in the control lines, which also discards DEGs that occur in lines of both TOF-01 and Father TOF-01 due to differences in the cell composition (i.e., based on similar mean differences in the percent of cTnT-positive cardiomyocytes).

Over-representation of gene ontologies and pathways. Over-representation analysis of gene ontology (GO) terms (molecular function, cellular component, biological process) and pathways (KEGG, Reactome) was performed using ConsensusPathDB-human (release 31), which is a meta-database of 32 public resources of biological interaction data comprising protein-protein, genetic, metabolic, signaling, gene regulatory and drug-target interactions in human[51]. To calculate the statistical significance of the overlap between candidate genes and

genes present in each reference GO term or pathway target list, a hypergeometric test was used. Genes with an adjusted $p<0.05$ (q-value) using the Benjamini-Hochberg method for controlling the FDR were defined as significantly enriched for the corresponding GO term or pathway.

Chromatin immunoprecipitation and qPCR. Chromatin immunoprecipitation (ChIP) experiments with iPSCs were performed using the MAGnify Chromatin Immunoprecipitation System (Thermo Fisher Scientific) according to the manufacturer's instructions with some modifications. Sonication was carried out in the Bioruptor pico (Diagenode) by applying 20 cycles of 30 sec ON and 30 sec OFF to obtain chromatin fragments of approximately 100-300 bp. For each ChIP, the p53 (DO-1) antibody (Santa Cruz) was applied. A mouse IgG ChIP was carried out as a negative control. The ChIP DNA was than purified using the ChIP DNA Clean & Concentrator kit (Zymo research). ChIP results were analyzed by qPCR. The P53 ChIP enrichment was normalized to the input and expressed as relative enrichment of the material precipitated by the indicated antibody on target regions.

Western blotting. Human iPSCs were treated with RIPA lysis buffer containing protease inhibitor (Roche) for protein isolation. Western blot was performed according to standard protocols. ImageJ was used to quantify the blot signals.

Linear mixed effect model. A linear mixed effect model was applied to investigate the impact of specific SNVs to transcriptional variation. As the aim was to determine whether an effect exists at the RNA level, all autosomal expressed protein-coding transcripts were filtered for regulatory SNVs called from WGS data. Regulatory SNVs were defined by overlapping with proximal promoter regions (i.e., 250 bp downstream from transcriptional state site), which also overlap transcription factor binding sites. For this subset of variations, those variations also present in the corresponding RNA-seq sample were selected. To calculate the fraction of variance explained by genotype, the transcriptional variation was decomposed into three components: (1) the genotype that was called in either the blood or iPSC sample (day 0) and validated in the corresponding RNAseq sample coded as a binary matrix, (2) the family affiliation that represents a possibly confounding factor also coded as a binary matrix; and (3) the differentiation stages (day 0, day 15, day 60). The normalized log TPM for a transcript *i* was modeled as a combination of two fixed effects and one normally distributed random effect, such that

$$Y_i = \beta_0 + \beta_1 GT_i + \beta_2\, Family_i + Z_1\, Diff.State_i + \varepsilon \quad (i = 1,\ldots,n),$$

where Y_i: gene expression as log(TPM) for all autosomal expressed protein-coding transcripts; β_0: intercept; $\beta_1\,GT_i$: coefficient for genotype called in WGS sample and validated in RNAseq; $\beta_2\,Family_i$: coefficient for family affiliation; Z_1 Diff. $State_i$: random variable for distinguishing the differentiation state of the sample (day 0, day 15 or day 60); ε: biological and technical error. The contribution of each coefficient is summarized by the fraction of variance explained for each transcript separately. All parameters were estimated with the maximum likelihood approach implemented in the lme4 package v1.1-12 in R. Variance estimates overall transcripts were calculated after parameter estimation via an expectation maximization approach based on the assumption that the variance estimates follow a gamma distribution.

Supplementary References

1. Cyganek, L. *et al.* Deep phenotyping of human induced pluripotent stem cell-derived atrial and ventricular cardiomyocytes. *JCI Insight.* **3,** (2018).

2. Wamstad, J. A. *et al.* Dynamic and Coordinated Epigenetic Regulation of Developmental Transitions in the Cardiac Lineage. *Cell.* **151,** 206 (2012).

3. Tohyama, S. *et al.* Distinct metabolic flow enables large-scale purification of mouse and human pluripotent stem cell-derived cardiomyocytes. *Cell Stem Cell.* **12,** 127–137 (2012).

4. Lian, X., Zhang, J., Azarin, S. M., Zhu, K. & Hazeltine, L. B. Directed cardiomyocyte differentiation from human pluripotent stem cells by modulating Wnt/β-catenin signaling under fully defined conditions. *Nat. Protoc.* **8,** 162-175 (2013).

5. Langmead, B. & Salzberg, S. L. Fast gapped-read alignment with Bowtie 2. *Nat. Methods.* **9,** 357–359 (2012).

6. McKenna, A. *et al.* The Genome Analysis Toolkit: a MapReduce framework for analyzing next-generation DNA sequencing data. *Genome Res.* **20,** 1297–1303 (2010).

7. Van der Auwera, G. A. *et al.* From FastQ data to high confidence variant calls: the Genome Analysis Toolkit best practices pipeline. *Curr. Protoc. Bioinformatics.* **43,** 11.10.1–33 (2014).

8. Cingolani, P. *et al.* A program for annotating and predicting the effects of single nucleotide polymorphisms, SnpEff: SNPs in the genome of Drosophila melanogaster strain w1118; iso-2; iso-3. *Fly (Austin).* **6,** 80–92 (2012).

9. Adzhubei, I., Jordan, D. M. & Sunyaev, S. R. Predicting functional effect of human missense mutations using PolyPhen-2. *Curr. Protoc. Hum. Genet.* **Chapter 7,** Unit7.20 (2013).

10. Kumar, P., Henikoff, S. & Ng, P. C. Predicting the effects of coding non-synonymous variants on protein function using the SIFT algorithm. *Nat. Protoc.* **4,** 1073–1081 (2009).

11. Schwarz, J. M., Cooper, D. N., Schuelke, M. & Seelow, D. MutationTaster2: mutation prediction for the deep-sequencing age. *Nat. Methods.* **11,** 361–362 (2014).

12. Boeva, V. *et al.* Control-FREEC: a tool for assessing copy number and allelic content using next-generation sequencing data. *Bioinformatics.* **28,** 423–425 (2011).

13. Chen, X. *et al.* Manta: rapid detection of structural variants and indels for germline and cancer sequencing applications. *Bioinformatics.* **32,** 1220–1222 (2015).

14. Harrow, J. *et al.* GENCODE: the reference human genome annotation for The ENCODE Project. *Genome Res.* **22,** 1760–1774 (2012).

15. Grunert, M. *et al.* Rare and private variations in neural crest, apoptosis and sarcomere genes define the polygenic background of isolated Tetralogy of Fallot. *Hum. Mol. Genet.* **23,** 3115–3128 (2014).

16. Kaynak, B. *et al.* Genome-wide array analysis of normal and malformed human hearts. *Circulation.* **107,** 2467–2474 (2003).

17. Toenjes, M. *et al.* Prediction of cardiac transcription networks based on molecular data and complex clinical phenotypes. *Mol. Biosyst.* **4,** 589–598 (2008).

18. Ricci, M., Xu, Y., Hammond, H. L. & Willoughby, D. A. Myocardial alternative RNA splicing and gene expression profiling in early stage hypoplastic left heart syndrome. *PLoS ONE.* **7,** e29784 (2012).

19. Schlesinger, J., Tönjes, M., Schueler, M. & Zhang, Q. Evaluation of the LightCycler® 1536 Instrument for high-throughput quantitative real-time PCR. *Methods.* **50,** S19-22 (2010).

20. Rodemoyer, A. *et al.* A tissue-specific gene expression template portrays heart development and pathology. *Hum. Genomics.* **8,** 6 (2014).

21. Sheng, W., Wang, H. & Ma, X. LINE-1 methylation status and its association with tetralogy of fallot in infants. *BMC Med. Genomics.* **5,** 20 (2012).

22. Grunert, M. *et al.* Comparative DNA methylation and gene expression analysis identifies novel genes for structural congenital heart diseases. *Cardiovasc. Res.* **112,** 464–477 (2016).

23. Sheng, W., Qian, Y. & Zhang, P. Association of promoter methylation statuses of congenital heart defect candidate genes with Tetralogy of Fallot. *J. Transl. Med.* **12,** 31 (2014).

24. Chowdhury, S., Erickson, S. W., MacLeod, S. L. & Cleves, M. A. Maternal genome-wide DNA methylation patterns and congenital heart defects. *PLoS ONE.* **6,** e16506 (2011).

25. Sheng, W., Qian, Y. & Wang, H. DNA methylation status of NKX2-5, GATA4 and HAND1 in patients with tetralogy of fallot. *BMC Med. Genomics.* **6,** 46 (2013).

26. Tomita-Mitchell, A. *et al.* Human gene copy number spectra analysis in congenital heart malformations. *Physiol. Genomics.* **44,** 518–541 (2012).

27. Warburton, D., Ronemus, M., Kline, J. & Jobanputra, V. The contribution of de novo and rare inherited copy number changes to congenital heart disease in an unselected sample of

children with conotruncal defects or hypoplastic left heart disease. *Hum. Genet.* **133,** 11 (2014).

28. Payne, A. R., Chang, S. W., Koenig, S. N., Zinn, A. R. & Garg, V. Submicroscopic chromosomal copy number variations identified in children with hypoplastic left heart syndrome. *Pediatr. Cardiol.* **33,** 757 (2012).

29. de Souza, K. R., Mergener, R., Huber, J., Pellanda, L. C. & Riegel, M. Cytogenomic Evaluation of Subjects with Syndromic and Nonsyndromic Conotruncal Heart Defects. *Biomed. Res. Int.* **2015,** 401941 (2015).

30. Geng, J. *et al.* Chromosome microarray testing for patients with congenital heart defects reveals novel disease causing loci and high diagnostic yield. *BMC Genomics.* **15,** 1127 (2014).

31. Bittel, D. C. *et al.* Ultra high-resolution gene centric genomic structural analysis of a non-syndromic congenital heart defect, Tetralogy of Fallot. *PLoS ONE.* **9,** e87472 (2014).

32. Greenway, S. C. *et al.* De novo copy number variants identify new genes and loci in isolated sporadic tetralogy of Fallot. *Nat. Genet.* **41,** 931–935 (2009).

33. Aguayo-Gómez, A. *et al.* Identification of Copy Number Variations in Isolated Tetralogy of Fallot. *Pediatr. Cardiol.* **36,** 1642–1646 (2015).

34. Hitz, M. P. & Lemieux, L. P. Rare copy number variants contribute to congenital left-sided heart disease. *PLoS Genet.* **8,** e1002903 (2012).

35. Erdogan, F. *et al.* High frequency of submicroscopic genomic aberrations detected by tiling path array comparative genome hybridisation in patients with isolated congenital heart disease. *J. Med. Genet.* **45,** 704–709 (2008).

36. Soemedi, R. *et al.* Phenotype-specific effect of chromosome 1q21.1 rearrangements and GJA5 duplications in 2436 congenital heart disease patients and 6760 controls. *Hum. Mol. Genet*. **21,** 1513–1520 (2011).

37. Wilson, K. D., Shen, P., Fung, E. & Karakikes, I. A rapid, high-quality, cost-effective, comprehensive and expandable targeted next-generation sequencing assay for inherited heart diseasesnovelty and significance. *Circ. Res.* **117,** 603 (2015).

38. Silversides, C. K. *et al.* Rare copy number variations in adults with tetralogy of Fallot implicate novel risk gene pathways. *PLoS Genet.* **8,** e1002843 (2012).

39. Soemedi, R. *et al.* Contribution of global rare copy-number variants to the risk of sporadic congenital heart disease. *Am. J. Hum. Genet.* **91,** 489–501 (2012).

40. Richards, A. A. *et al.* Cryptic chromosomal abnormalities identified in children with congenital heart disease. *Pediatr. Res.* **64,** 358–363 (2008).

41. Bansal, V. *et al.* Outlier-based identification of copy number variations using targeted resequencing in a small cohort of patients with Tetralogy of Fallot. *PLoS ONE.* **9,** e85375 (2014).

42. Andersen, T. A., Troelsen, K. & Larsen, L. A. Of mice and men: molecular genetics of congenital heart disease. *Springer*. **71,** 1327 (2014).

43. Rickert-Sperling, S., Kelly, R. G. & Driscoll, D. J. Congenital Heart Diseases: The Broken Heart. Clinical Features, Human Genetics and Molecular Pathways. *Springer.* (2016).

44. O'Brien, J. E. *et al.* Noncoding RNA expression in myocardium from infants with tetralogy of Fallot. *Circ. Cardiovasc. Genet.* **5,** 279–286 (2012).

45. Sucharov, C. C. *et al.* Micro-RNA expression in hypoplastic left heart syndrome. *J. Card. Fail.* **21,** 83–88 (2014).

46. Wei, C.-L. *et al.* A global map of p53 transcription-factor binding sites in the human genome. *Cell.* **124,** 207–219 (2006).

47. Akdemir, K. C. *et al.* Genome-wide profiling reveals stimulus-specific functions of p53 during differentiation and DNA damage of human embryonic stem cells. *Nucleic. Acids. Res.* **42,** 205–223 (2013).

48. Dobin, A. *et al.* STAR: ultrafast universal RNA-seq aligner. *Bioinformatics.* **29,** 15–21 (2012).

49. Li, B. & Dewey, C. N. RSEM: accurate transcript quantification from RNA-Seq data with or without a reference genome. *BMC Bioinformatics.* **12,** 323 (2011).

50. Robinson, M. D., McCarthy, D. J. & Smyth, G. K. edgeR: a Bioconductor package for differential expression analysis of digital gene expression data. *Bioinformatics.* **26,** 139–140 (2009).

51. Herwig, R., Hardt, C., Lienhard, M. & Kamburov, A. Analyzing and interpreting genome data at the network level with ConsensusPathDB. *Nat. Protoc.* **11,** 1889–1907 (2016).

Supplementary Figures

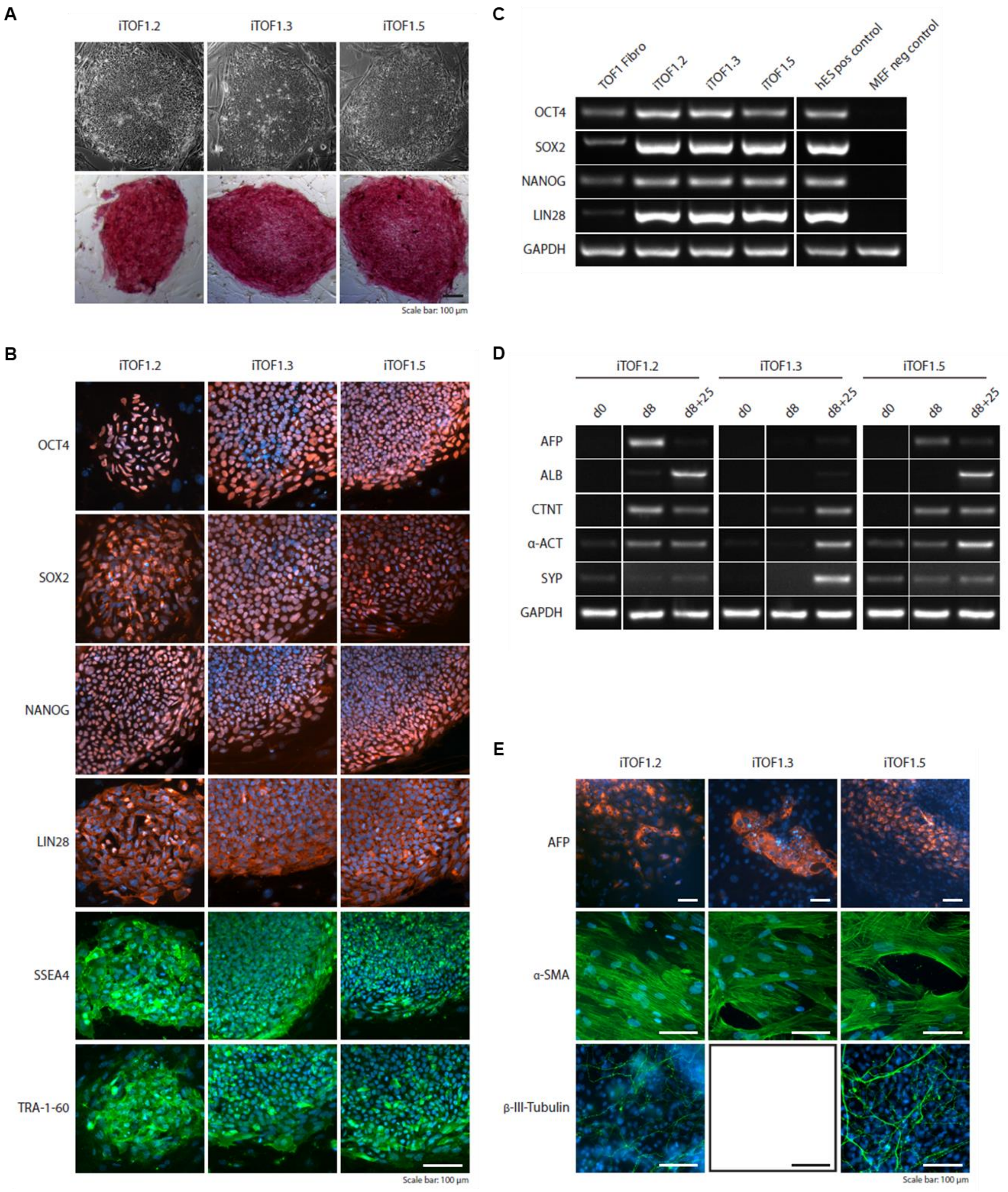

Supplementary Figure S1. Characterization of the generated iPSC clones of TOF-01. (A) Morphology and alkaline phosphatase staining of the generated iPSC colonies. (B) Immunofluorescence analysis of pluripotency markers. DNA was stained with DAPI. (C) Gene expression analysis of pluripotency markers using RT-PCR. GAPDH was used as a reference gene. (D) Gene expression analysis of germlayer markers using RT-PCR. GAPDH was used as a reference gene. (C-D) The grouping of blots was cropped from different parts of the same

gel, or from different gels. Uncropped blots are available in Supplementary Fig. S6 and S7, respectively. (E) Immunofluorescence analysis of germlayer markers. Scale bar for all representative sections 100µm. DNA was stained with DAPI.

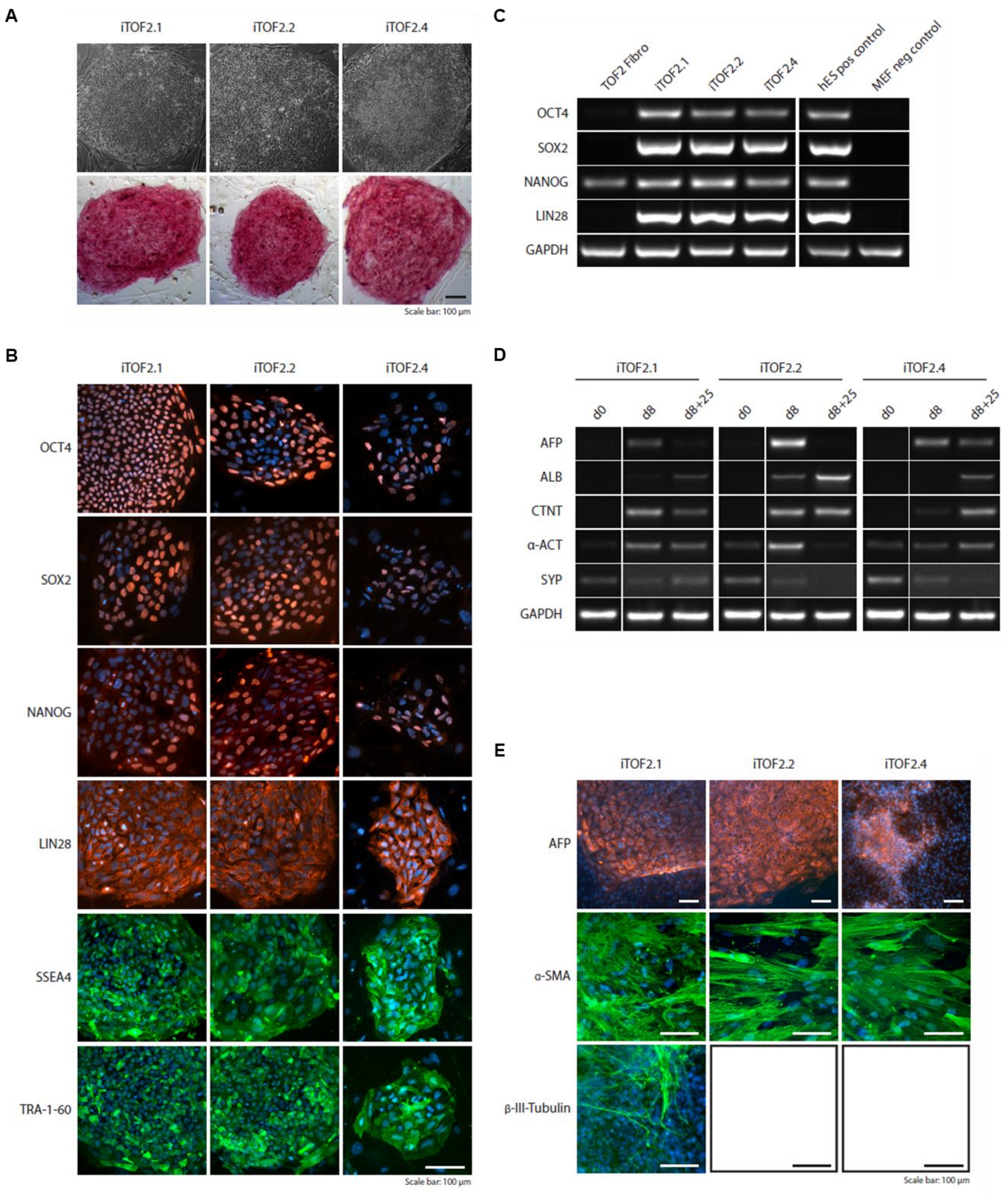

Supplementary Figure S2. Characterization of the generated iPSC clones of Father TOF-01. (A) Morphology and alkaline phosphatase staining of the generated iPSC colonies. (B) Immunofluorescence analysis of pluripotency markers. DNA was stained with DAPI. (C) Gene expression analysis of pluripotency markers using RT-PCR. GAPDH was used as a reference gene. (D) Gene expression analysis of germlayer markers using RT-PCR. GAPDH was used as a reference gene. (C-D) The grouping of blots was cropped from different parts of the same gel, or from different gels. Uncropped blots are available in Supplementary Fig. S6 and S7, respectively. (E) Immunofluorescence analysis of germlayer markers. Scale bar for all representative sections 100µm. DNA was stained with DAPI.

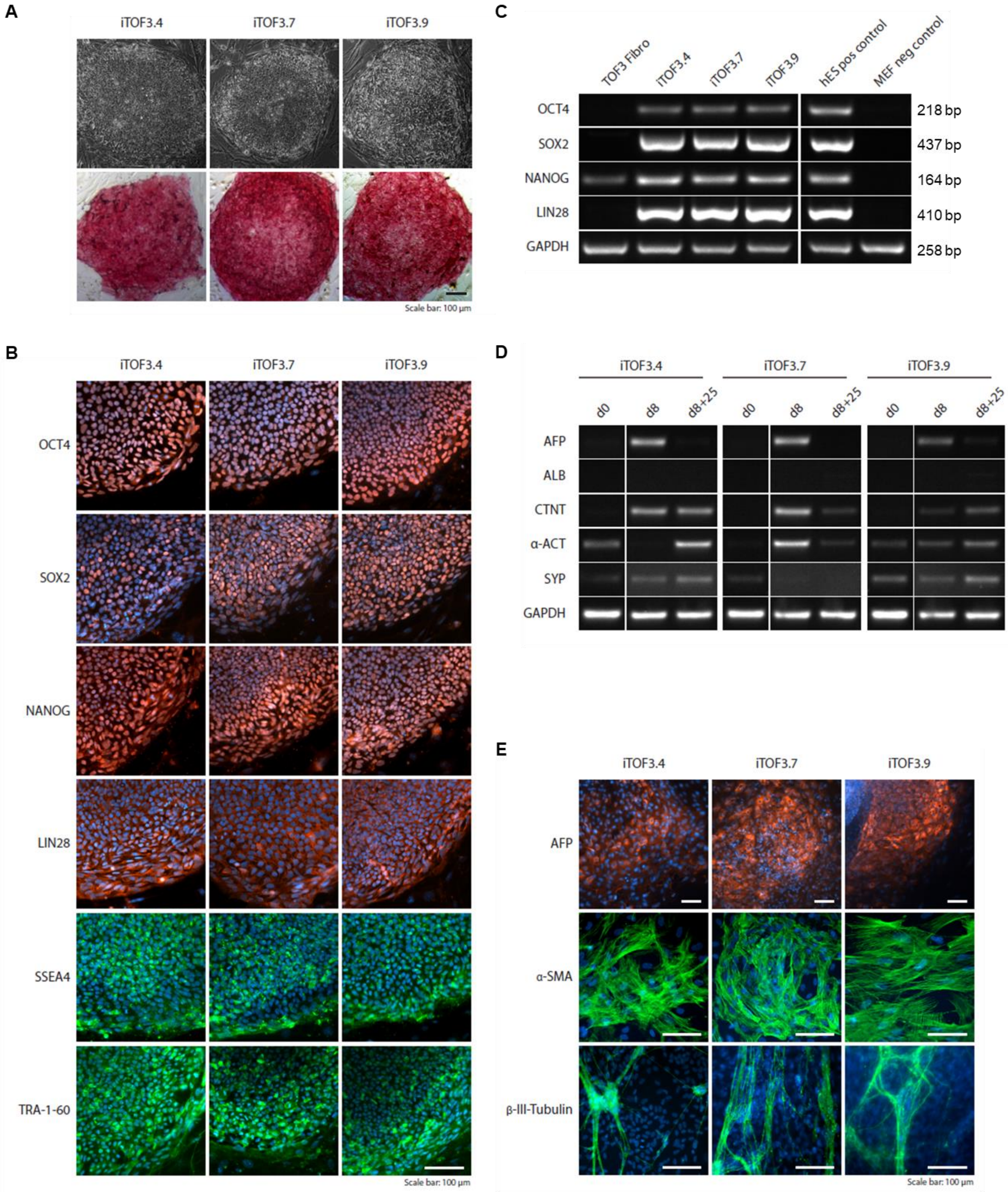

Supplementary Figure S3. Characterization of the generated iPSC clones of Sister TOF-01. (A) Morphology and alkaline phosphatase staining of the generated iPSC colonies. (B) Immunofluorescence analysis of pluripotency markers. DNA was stained with DAPI. (C) Gene expression analysis of pluripotency markers using RT-PCR. GAPDH was used as a reference gene. (D) Gene expression analysis of germlayer markers using RT-PCR. GAPDH was used as a reference gene. (C-D) The grouping of blots was cropped from different parts of the same gel, or from different gels. Uncropped blots are available in Supplementary Fig. S6 and S7, respectively. (E) Immunofluorescence analysis of germlayer markers. Scale bar for all representative sections 100µm. DNA was stained with DAPI.

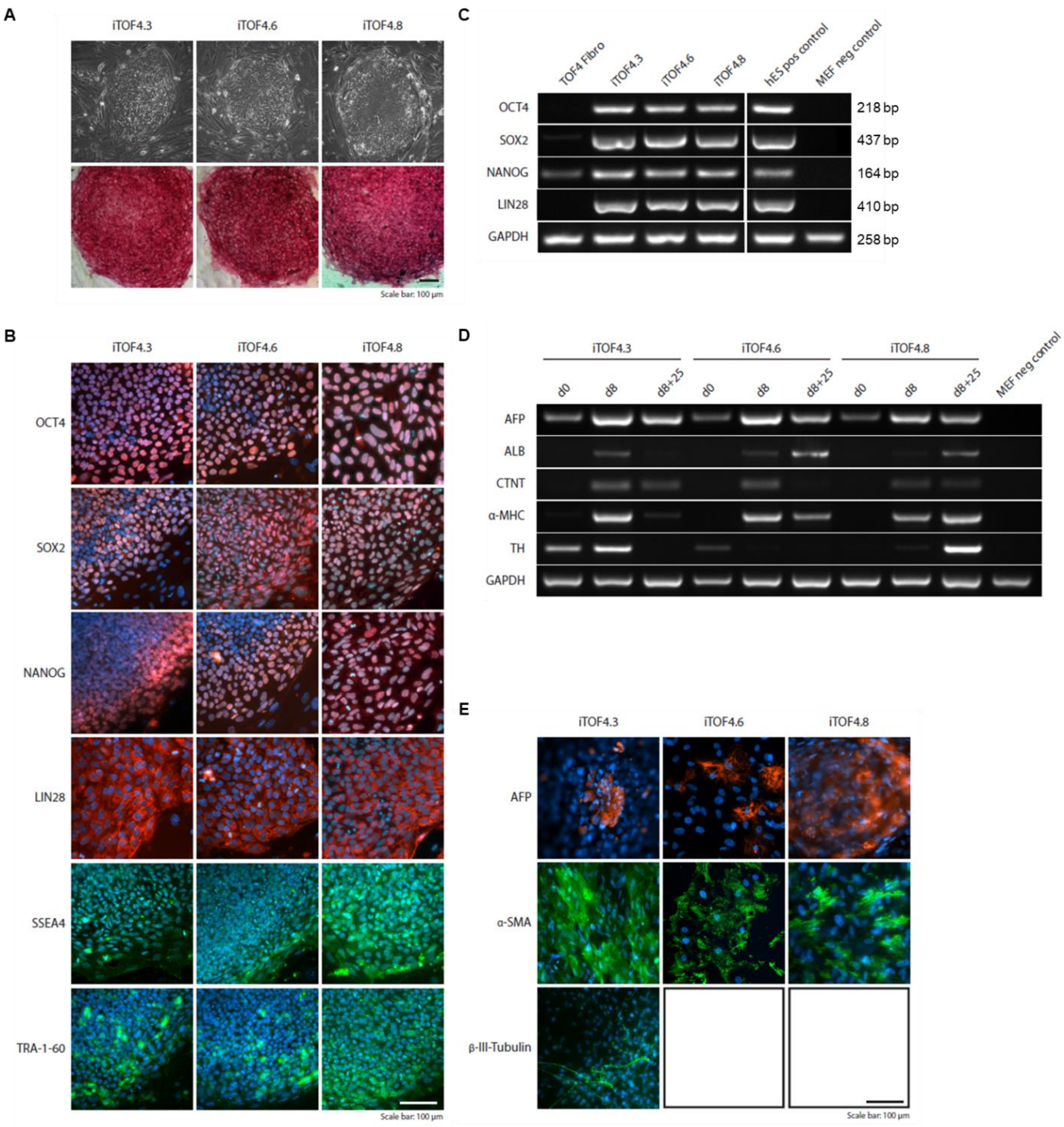

Supplementary Figure S4. Characterization of the generated iPSC clones of Mother TOF-02. (A) Morphology and alkaline phosphatase staining of the generated iPSC colonies. (B) Immunofluorescence analysis of pluripotency markers. DNA was stained with DAPI. (C) Gene expression analysis of pluripotency markers using RT-PCR. GAPDH was used as a reference gene. (D) Gene expression analysis of germlayer markers using RT-PCR. GAPDH was used as a reference gene. (C-D) The grouping of blots was cropped from different parts of the same gel, or from different gels. Uncropped blots are available in Supplementary Fig. S6 and S8, respectively. (E) Immunofluorescence analysis of germlayer markers. Scale bar for all representative sections 100µm. DNA was stained with DAPI.

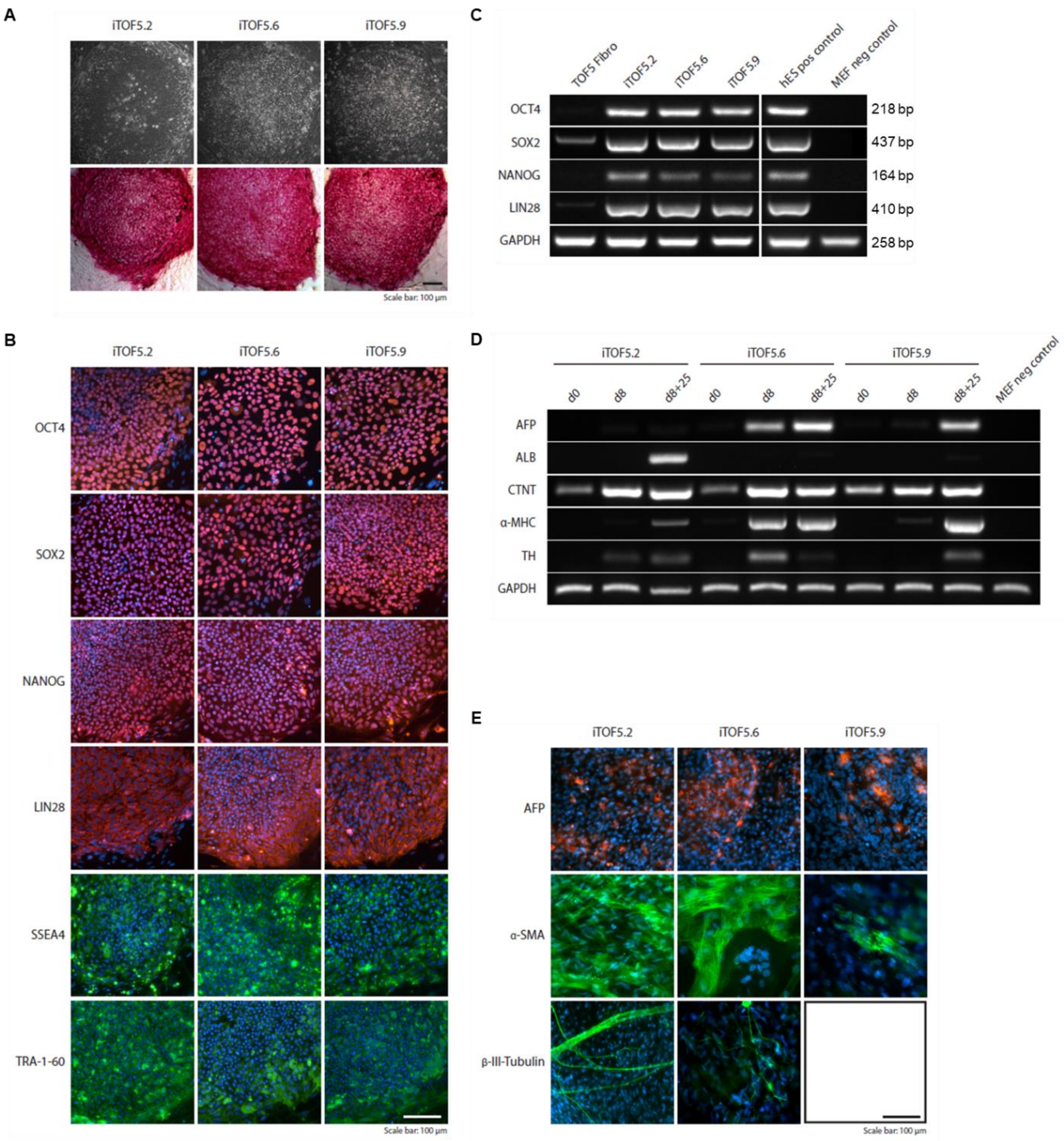

Supplementary Figure S5. Characterization of the generated iPSC clones of TOF-02. (A) Morphology and alkaline phosphatase staining of the generated iPSC colonies. (B) Immunofluorescence analysis of pluripotency markers. DNA was stained with DAPI. (C) Gene expression analysis of pluripotency markers using RT-PCR. GAPDH was used as a reference gene. (D) Gene expression analysis of germlayer markers using RT-PCR. GAPDH was used as a reference gene. (C-D) The grouping of blots was cropped from different parts of the same gel, or from different gels. Uncropped blots are available in Supplementary Fig. S6 and S8, respectively. (E) Immunofluorescence analysis of germlayer markers. Scale bar for all representative sections 100µm. DNA was stained with DAPI.

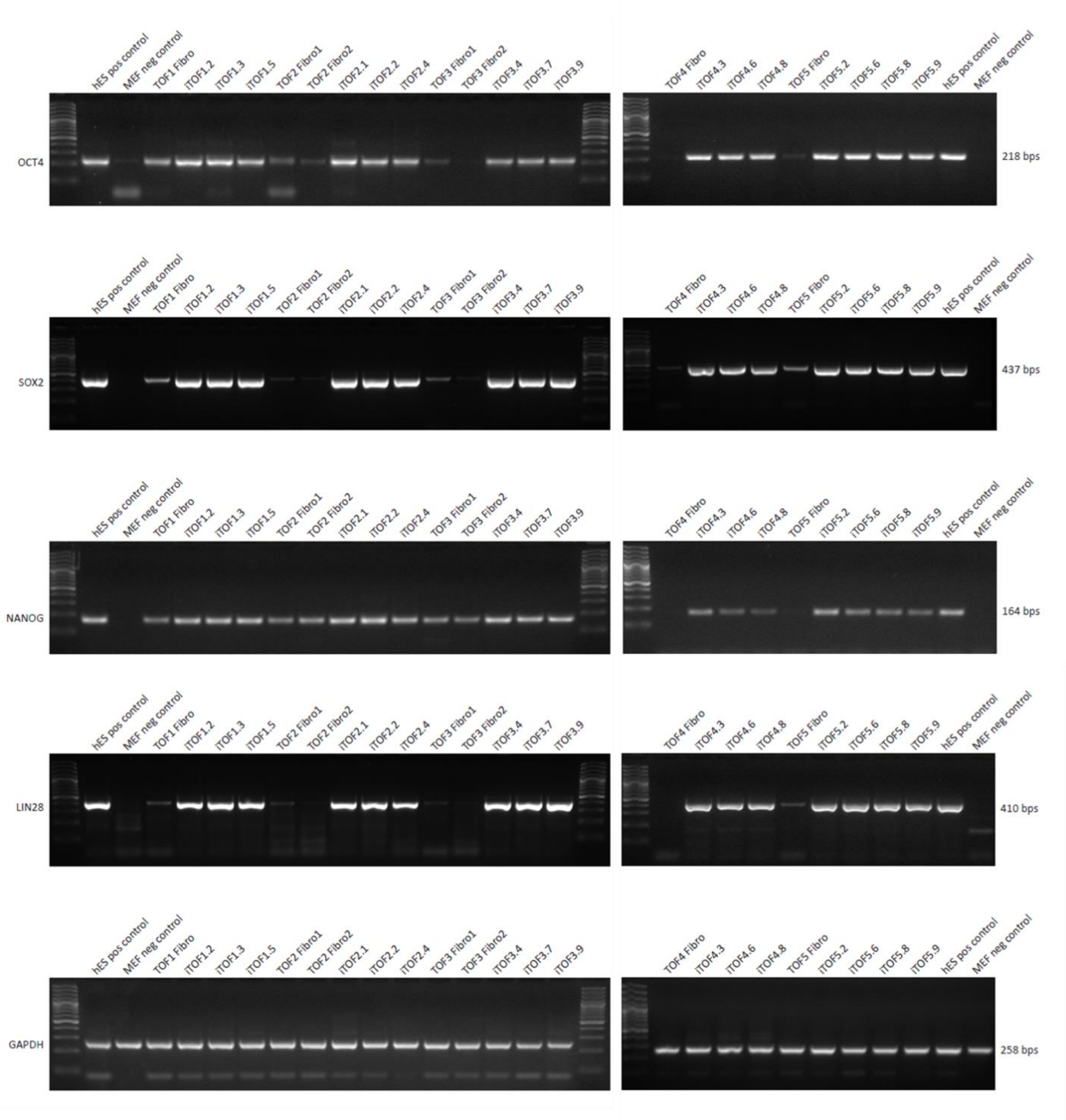

Supplementary Figure S6. Gene expression analysis of pluripotency markers using RT-PCR. GAPDH was used as a reference gene. Uncropped blots related to Supplementary Fig. S1C, S2C, S3C, S4C, and S5C.

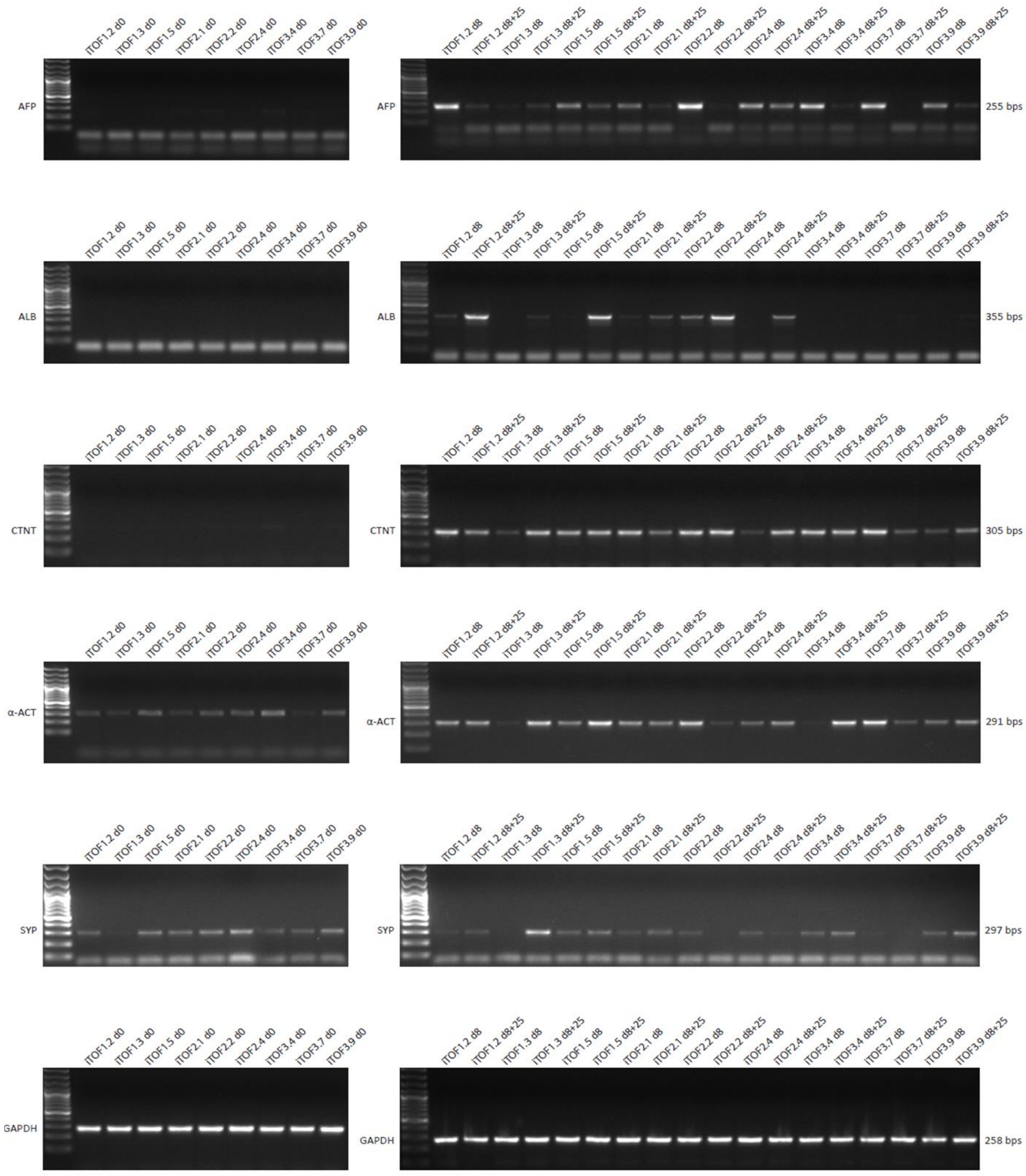

Supplementary Figure S7. Gene expression analysis of pluripotency markers using RT-PCR. GAPDH was used as a reference gene. Uncropped blots related to Supplementary Fig. S1D, S2D, and S3D.

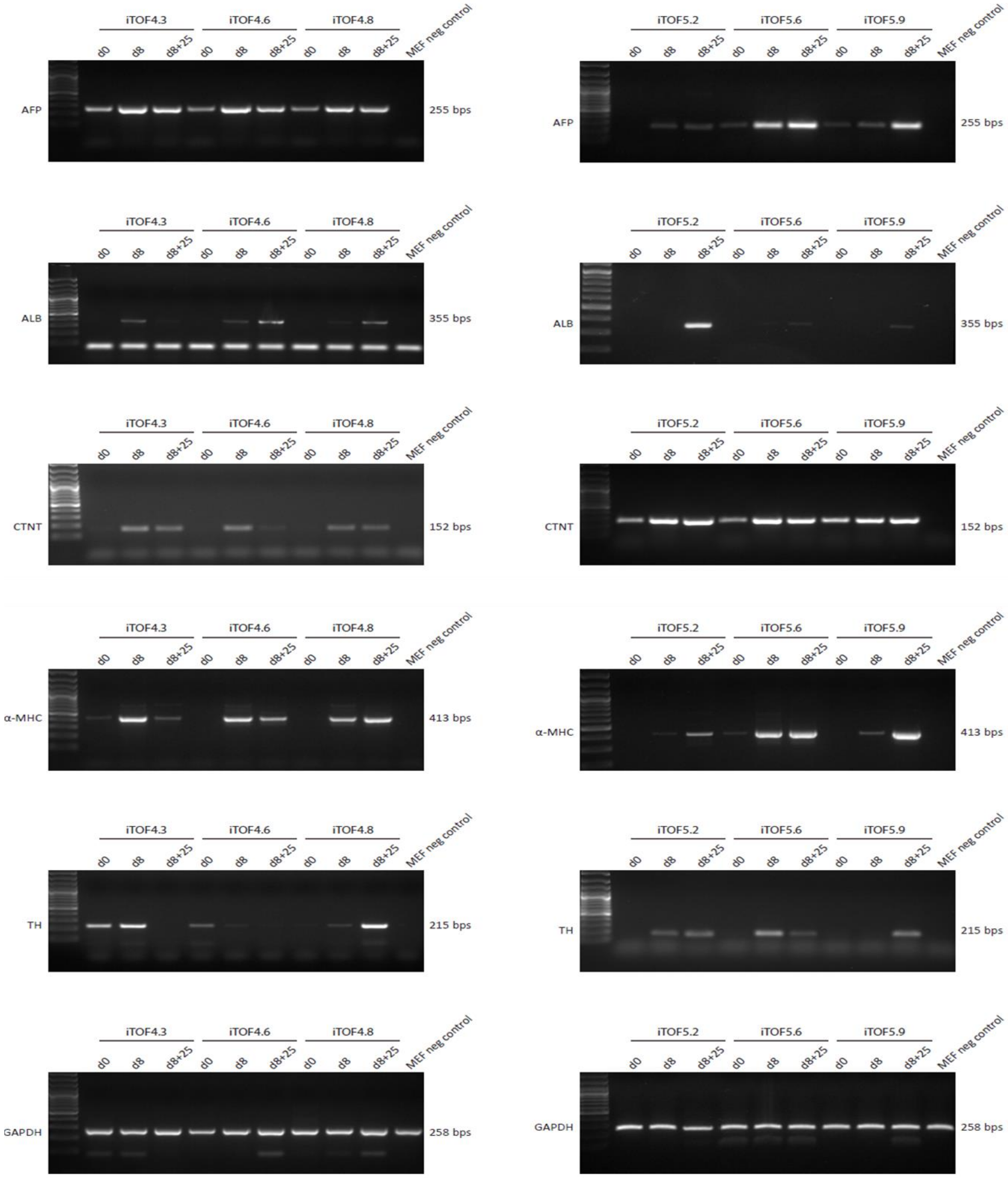

Supplementary Figure S8. Gene expression analysis of pluripotency markers using RT-PCR. GAPDH was used as a reference gene. Uncropped blots related to Supplementary Fig. S4D and S5D.

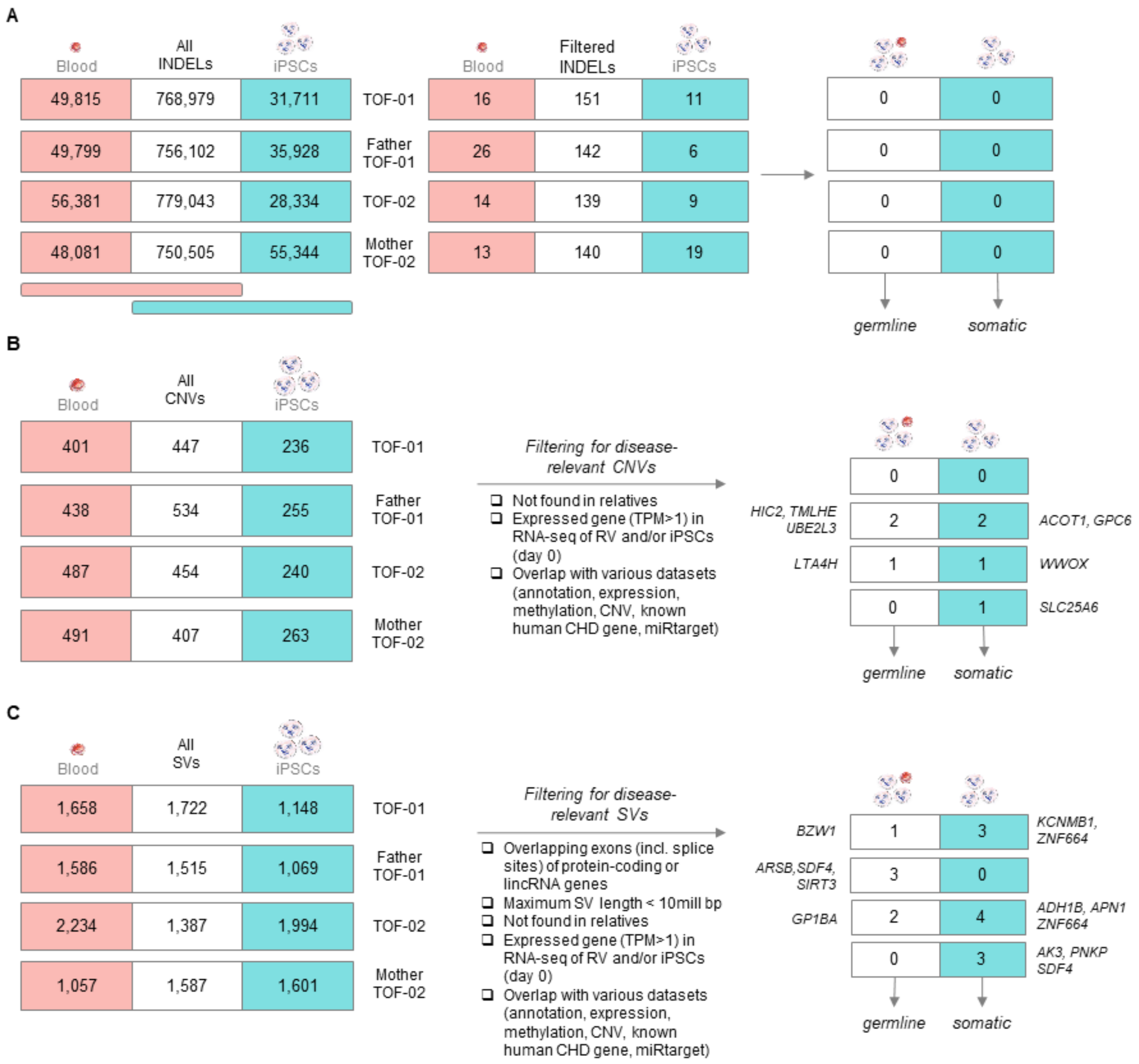

Supplementary Figure S9. Identified candidate genes with disease-relevant somatic and/or germline insertions and deletions (INDELs), copy number variations (CNVs) and structural variations (SVs) in pooled iPSCs (n=3) of TOF patients and healthy relatives. (A) INDELs identified in blood and/or iPSCs and filtering for disease candidate genes with rare and damaging variations. (B) CNVs identified and filtered for exclusive CNVs located on expressed genes (TPM>1) overlapping disease-relevant gene datasets. (C) SVs identified and filtered for exclusive SVs overlapping in coding regions of expressed disease-relevant genes.

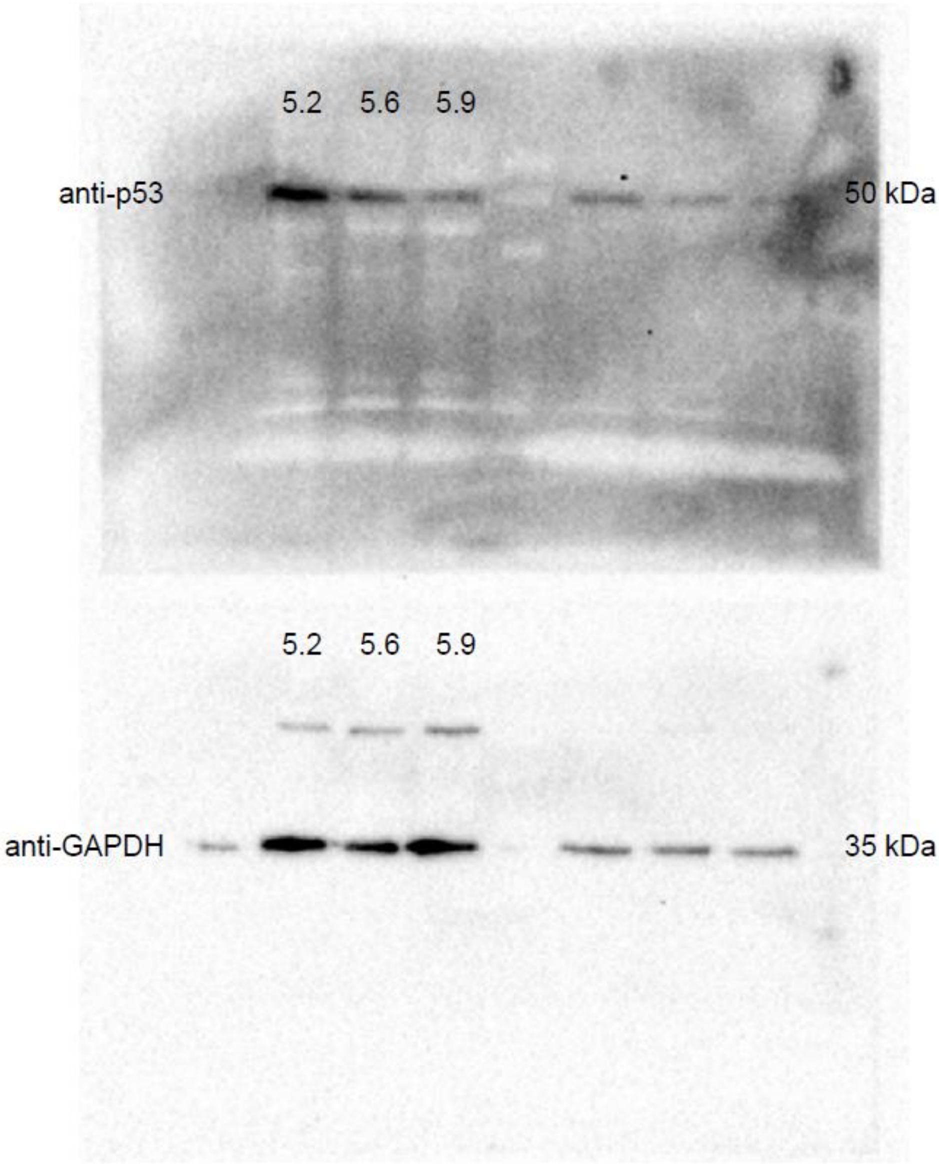

Supplementary Figure S10. The protein level of P53 in the individual iPSC clones of TOF-02. GAPDH was used as the internal control. Uncropped gels/blots related to Fig. 3D.

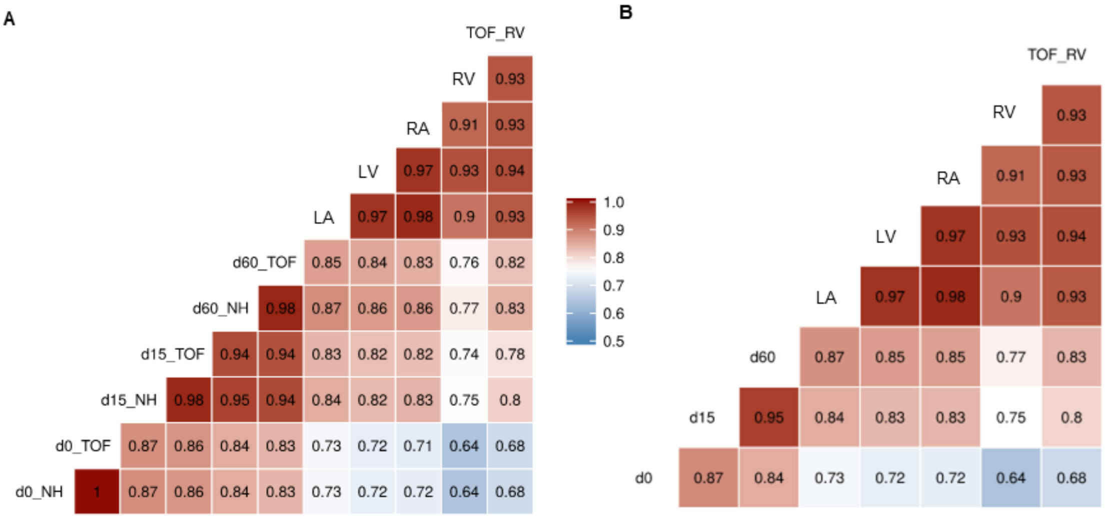

Supplementary Figure S11. Correlation to healthy and TOF heart tissue. (A) Median expression of iPSCs and derived CMs for TOF patients and healthy individuals separately versus median heart tissue expression. Plots were generated with the R function *ggcorr* with an adapted gradient range of 50 to 100 (midpoint 75). The correlation is based on pairwise spearman correlation. (B) Combined TOF patient and healthy individual's median expression of iPSCs (n=15, i.e. n=6 for TOF & n=9 for NH) and derived CMs (n=7 at d15, i.e., n=2 for TOF and n=5 for NH; and n=9 at d60, i.e., n=3 for TOF and n=6 for NH) versus median heart tissue expression for left ventricle (LV; n=4), left atrium (LA; n=4), right ventricle (RV; n=4), right atrium (RA; n=4) of healthy hearts and TOF right ventricle (TOF_RV; n=8). Plots were generated with the R function *ggcorr* with an adapted gradient range of 50 to 100 (midpoint 75). The correlation is based on pairwise spearman correlation.

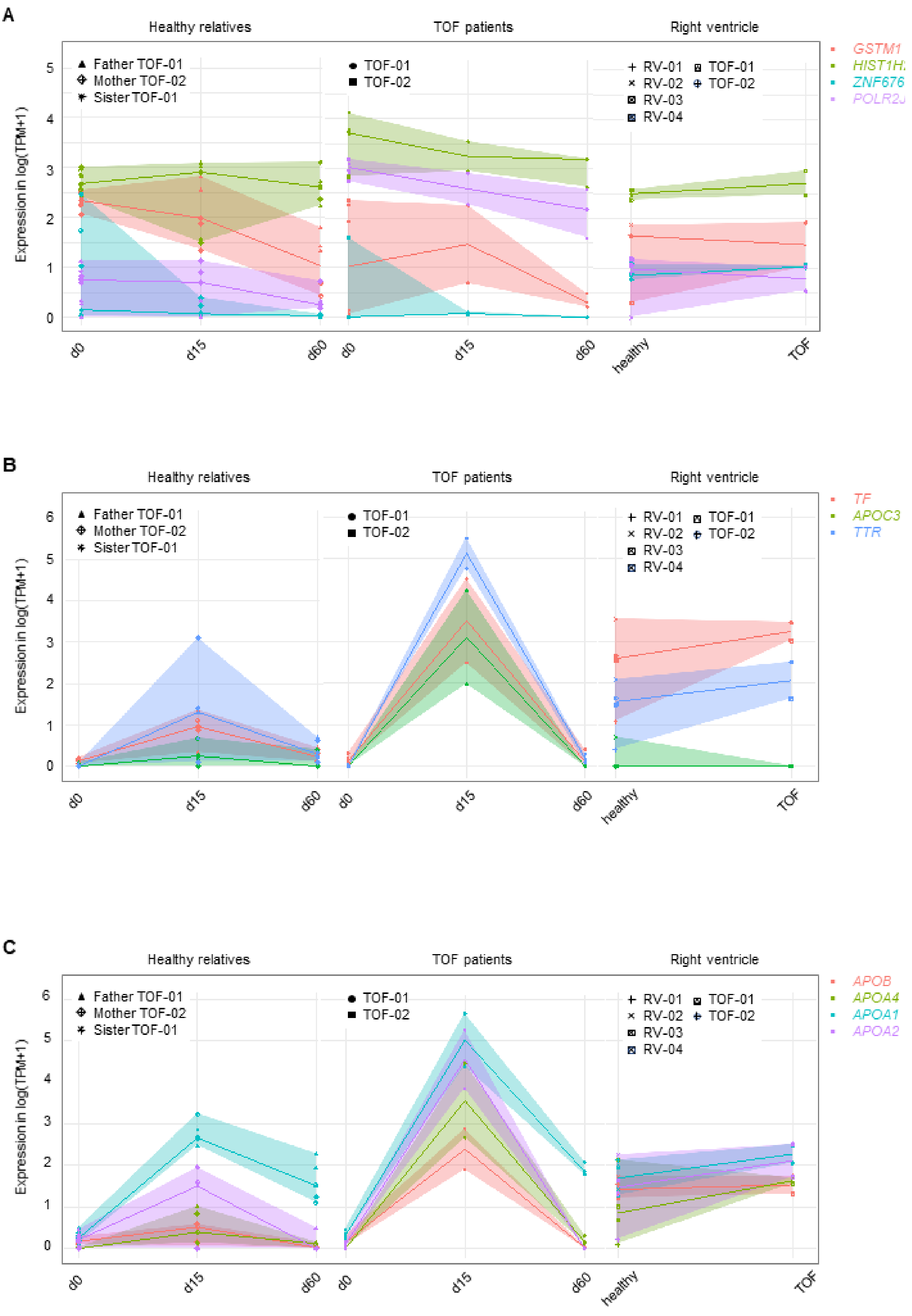

Supplementary Figure S12. Expression of differentially expressed genes in iPSCs at day 0 and cardiomyocytes at day 15 between TOF patients and healthy relatives. (A) Expression of

genes differentially expressed at day 0. (B-C) Expression of genes differentially expressed at day 15. Gene expression is depicted at day 0, day 15 and day 60 for healthy relatives (left) and TOF patients (center) of each clone (n=3 for each individual except CMs at d15 with n=2 for TOF-01 and Father TOF-01; n=0 for TOF-02 & sister TOF-01 at d15 & d60) as well as for right ventricle of healthy hearts (RV) individuals and TOF-01 and TOF-02 in the right ventricle (right). The TPMs (Transcripts Per Kilobase Million) are log scaled.

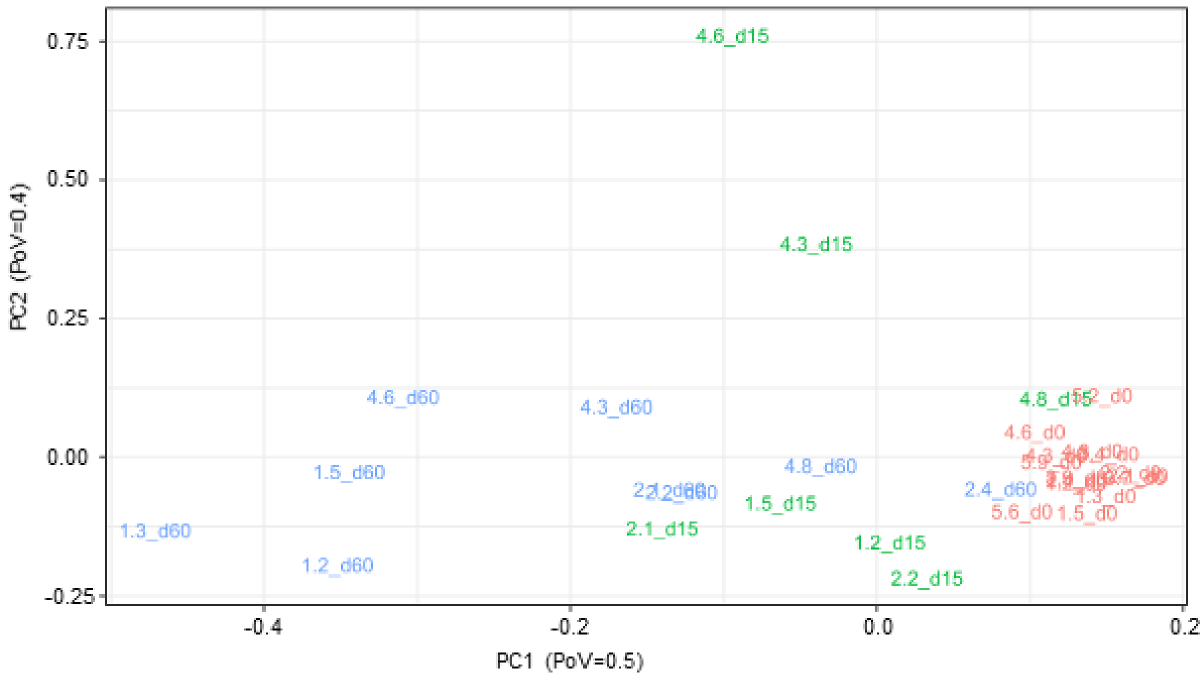

Supplementary Figure S13. Principle component analysis (PCA) for RNA-seq samples at day 0 (n=12), day 15 (n=7) and day 60 (n=9). PCA is based on TPM (Transcripts Per Kilobase Million) values of genes of at least one TPM in one sample. Sample IDs are based on the clone ID and the stage of differentiation (day 0: red; day 15: green; day 60: blue).

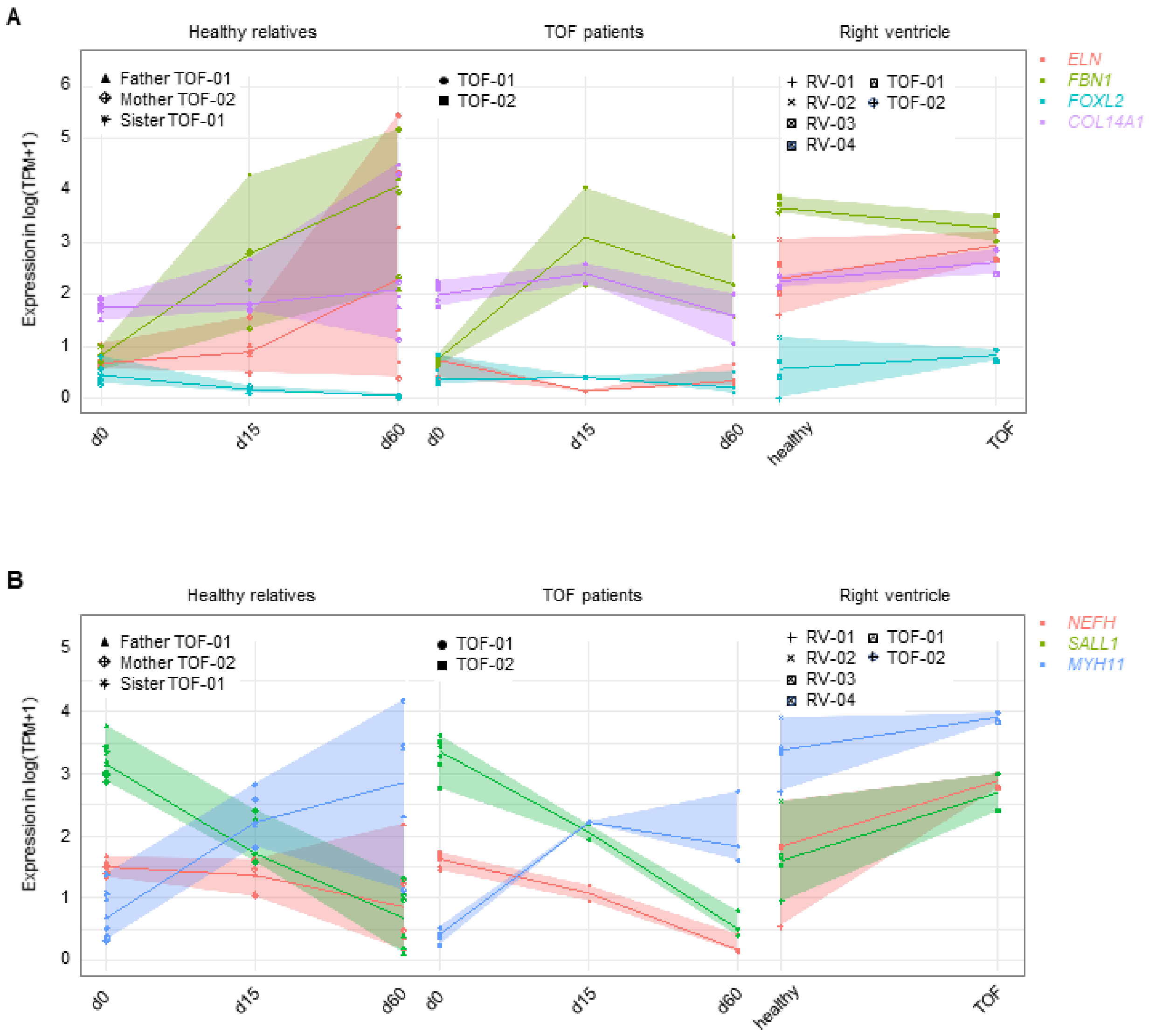

Supplementary Figure S14. Expression of differentially expressed genes at day 60 between patients and healthy parent. Gene expression is depicted over day 0, day 15, day 60 for healthy relatives (left) and TOF patients (center) for each clone (n=3 for each individual except CMs at d15 with n=2 for TOF-01 and Father TOF-01; n=0 for TOF-02 & sister TOF-01 at d15 & d60) as well as for right ventricle of healthy hearts (RV) individuals and TOF-01 and TOF-02 in the right ventricle (right). The TPM (Transcripts Per Kilobase Million) values are log scaled.

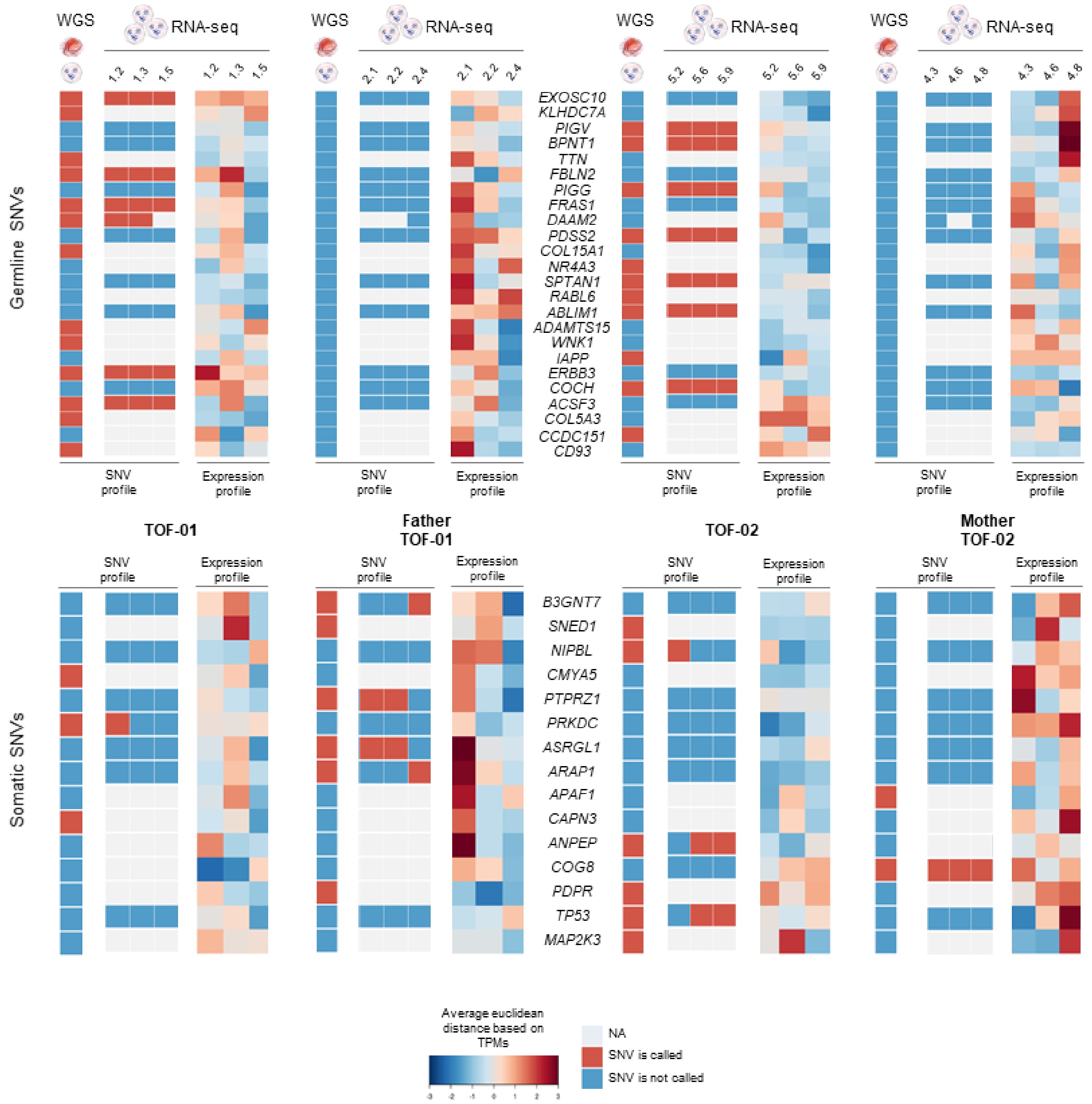

Supplementary Figure S15. Expression profiles of genes with rare damaging germline single nucleotide variations (SNVs) in patients and somatic SNVs in either patient or healthy parent. Profiles are divided into genes containing germline SNVs (top) and somatic SNVs (bottom) as well as in family 1 (left) and family 2 (right). The SNV status in whole genome sequencing (WGS) of the pooled iPSCs (n=3) for each gene is always shown left for each individual (red: SNV called, blue: SNV not called). The status in RNA-seq of the SNVs called in WGS is divided into the heatmap of expression (right) and the SNV status in the RNA-seq data for each individual clone (gray: coverage insufficient). A SNV is marked as called in RNA-seq if more or equal to 10 reads overlap the position and the SNV is present in at least 20% or in at least 5 reads.

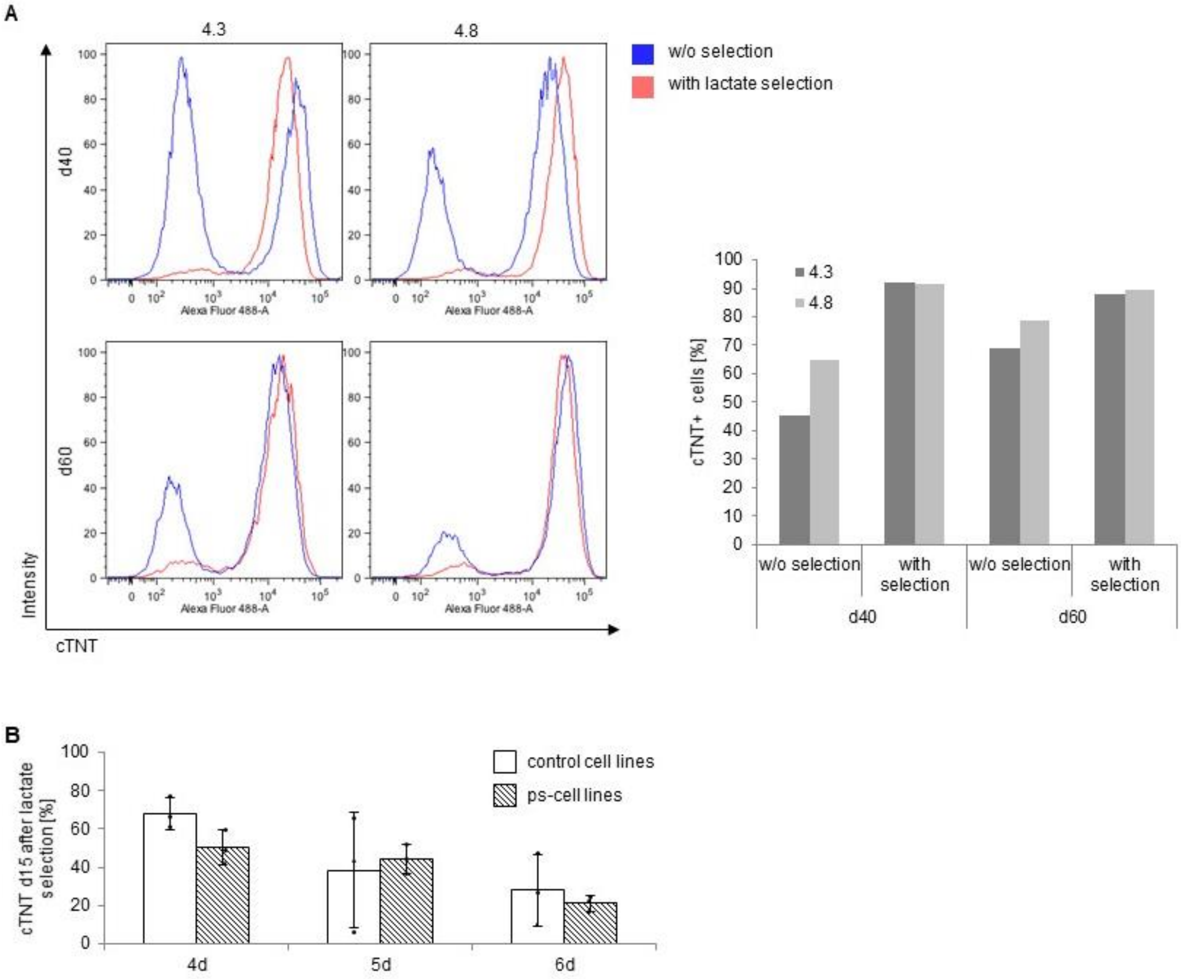

Supplementary Figure S16. Differentiation efficiency with or without lactate selection. (A) Lactate selection gave rise to ~90 % cardiomyocytes (91% at day 40 and 89% at day 60). Efficiency of lactate selection was not dependent on initial differentiation efficiency. With increasing culture period, a decreasing number of non-cardiomyocytes survive (n=1 for clone 4.1 and 4.8). (B) Differentiation efficiency after day 15 using lactate-supplemented media (n=3 for each state and line).

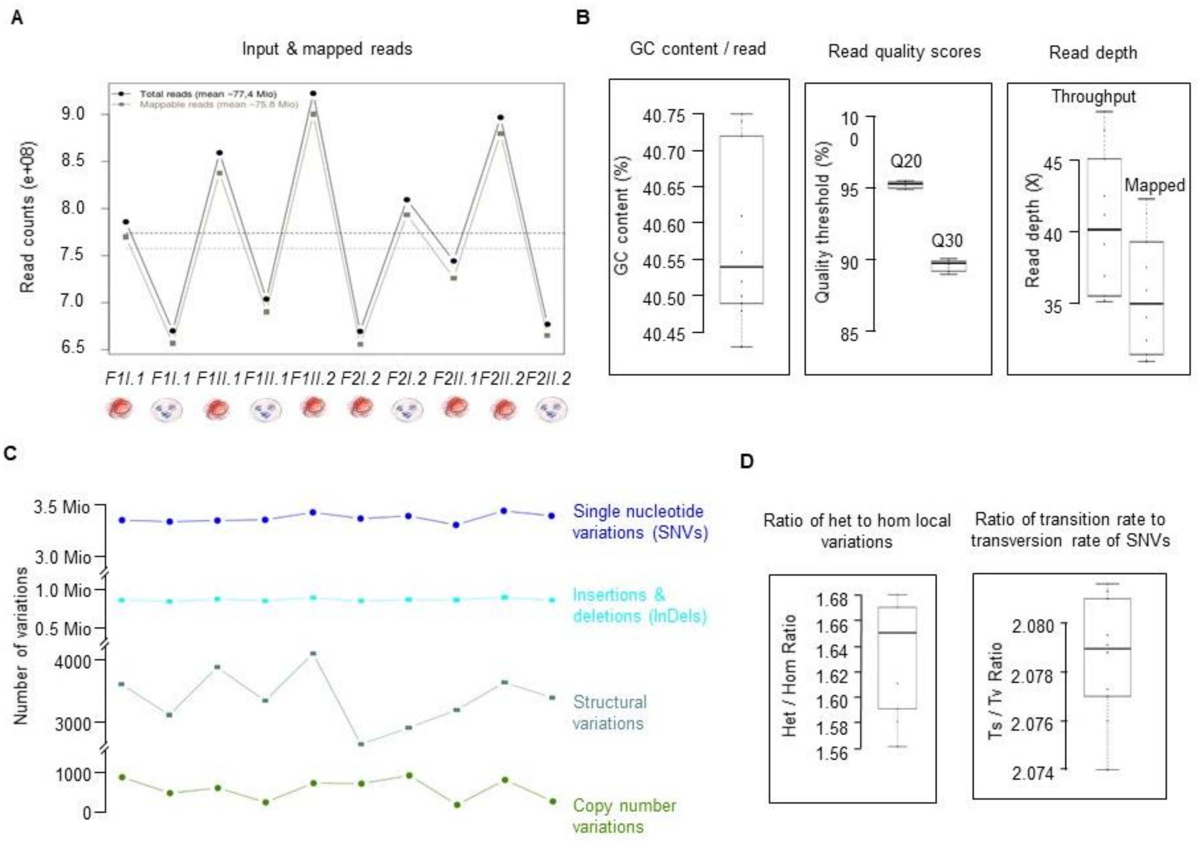

Supplementary Figure S17. Whole genome sequencing results and quality control. (A) Overview of the number of input and mapped reads for all sequenced blood and pooled iPSC (n=3) samples (in total n=10). (B) Ranges over all samples for GC content, read quality score and read depth. (C) Number of single nucleotide variations (SNVs), insertion and deletion (INDELs), structural variation (SVs) and copy number variations (CNVs) per sample. (D) Ratio of heterozygous and homozygous variations after SNV calling as well as transition to transversion rate.

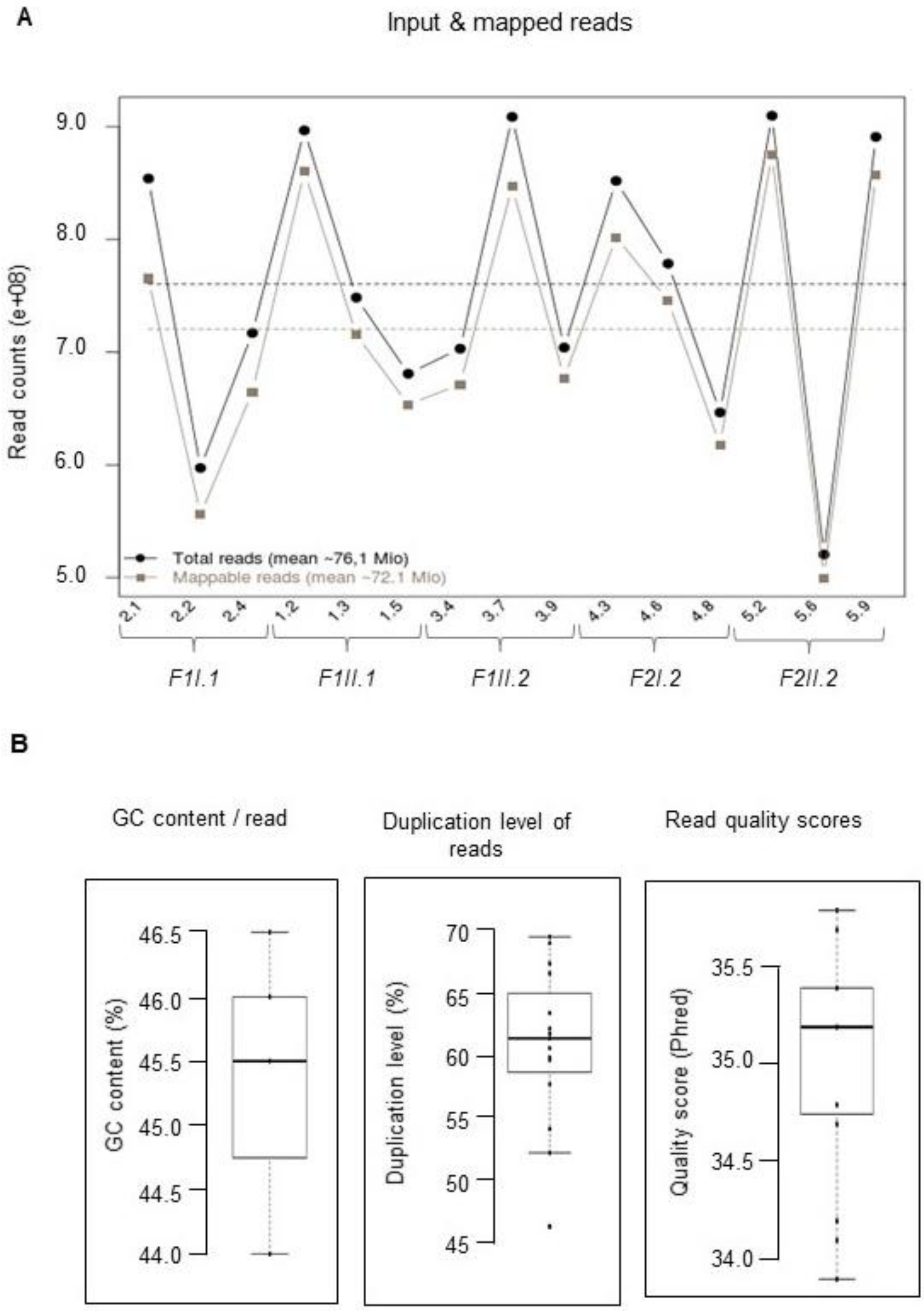

Supplementary Figure S18. RNA sequencing results and quality control. (A) Overview of the number of input and mapped reads for all clones (n=15). (B) Ranges of GC content, duplication level and read quality scores for input reads over all clones. The input reads show an average GC content of 45.5%, the duplication rate is 62% and the average quality score is 35.3 (Phred score).
